# Electrolyte‐Wettability Issues and Challenges of Electrode Materials in Electrochemical Energy Storage, Energy Conversion, and Beyond

**DOI:** 10.1002/advs.202300283

**Published:** 2023-04-21

**Authors:** Lei Zhao, Yuan Li, Meimei Yu, Yuanyou Peng, Fen Ran

**Affiliations:** ^1^ State Key Laboratory of Advanced Processing and Recycling of Non‐ferrous Metals Department of Polymeric Materials Science and Engineering School of Materials Science and Engineering Lanzhou University of Technology Lanzhou Gansu 730050 P. R. China

**Keywords:** electrode materials, electrode/electrolyte interface, electrolyte‐wettability, energy conversion, energy storage

## Abstract

The electrolyte‐wettability of electrode materials in liquid electrolytes plays a crucial role in electrochemical energy storage, conversion systems, and beyond relied on interface electrochemical process. However, most electrode materials do not have satisfactory electrolyte‐wettability for possibly electrochemical reaction. In the last 30 years, there are a lot of literature have directed at exploiting methods to improve electrolyte‐wettability of electrodes, understanding basic electrolyte‐wettability mechanisms of electrode materials, exploring the effect of electrolyte‐wettability on its electrochemical energy storage, conversion, and beyond performance. This review systematically and comprehensively evaluates the effect of electrolyte‐wettability on electrochemical energy storage performance of the electrode materials used in supercapacitors, metal ion batteries, and metal‐based batteries, electrochemical energy conversion performance of the electrode materials used in fuel cells and electrochemical water splitting systems, as well as capacitive deionization performance of the electrode materials used in capacitive deionization systems. Finally, the challenges in approaches for improving electrolyte‐wettability of electrode materials, characterization techniques of electrolyte‐wettability, as well as electrolyte‐wettability of electrode materials applied in special environment and other electrochemical systems with electrodes and liquid electrolytes, which gives future possible directions for constructing interesting electrolyte‐wettability to meet the demand of high electrochemical performance, are also discussed.

## Graphical abstract

The electrolyte‐wettability of electrode materials has remarkable impact on their electrochemical performance. This review elucidates the basic electrolyte‐wettability mechanisms of electrode materials, provides a comprehensive evaluation of the topic by summarizing recent progress in the research of electrolyte‐wettability of electrode in electrochemical energy storage systems, energy conversion systems, and capacitive deionization, and proposes critical issues, challenges, and perspectives.

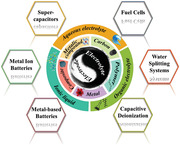

## 1 Introduction

There are enormous literature describe the research over the past 60 years that have directed at studying and/or controlling the interaction between solid and liquid.^[^ ^1^ ^]^ The wetting behavior of liquid toward solid surfaces is a very important aspect of the research mentioned above, which may have a wide variety of practical applications in daily life, industry, and agriculture.^[^ ^2^, ^3^, ^4^ ^]^ In some fields, the solid surface is expected to be less wetted by liquid, for instance, cookware coatings,^[^ ^5^ ^]^ self‐cleaning window for the industry of automotive and aeronautics,^[^ ^6^ ^]^ waterproof textile,^[^ ^7^ ^]^ antifingerprint,^[^ ^8^ ^]^ and so on. By contrary, in some specific fields, the more excellent wettability of solid surface by liquid is required to satisfy some needs that could improve antireflective properties for optical instruments and mobile phones,^[^ ^9^ ^]^ liquid transportation,^[^ ^10^ ^]^ membrane separation,^[^ ^11^ ^]^ cell^[^ ^12^ ^]^ and antibacterial adhesion,^[^ ^13^ ^]^ solid lubricity,^[^ ^14^ ^]^ and combination property of the applied function materials.^[^ ^15^ ^]^ Especially, in the fields based on interfacial interactions, the wettability of solid surface by liquid is one of the most critical factors, because it directly determines whether the interfacial interaction could happen and how much and how efficiently the interfacial interaction happen.^[^ ^16^, ^17^ ^]^ Inferior wetting and even no wetting in solid/liquid interface results in poor interaction degree and interaction efficiency, and even no interaction; whereas, superior wetting gives rise to a more efficient interfacial interaction. Thus, the wettability in the solid/liquid interface plays a crucial role in electrochemical energy storage, energy conversion, and beyond systems relied on interfacial interaction.

In the 21st century, one of the great challenges is unquestionably the research and development of energy storage and conversion devices, in order to cope with the potential exhaustion of fossil fuels and environmental pollution.^[^ ^18^, ^19^ ^]^ Electrochemical energy storage and conversion relied on interfacial interaction is under serious consideration as an alternative energy/power source, ascribing to its more sustainable and more environmentally friendly designability.^[^ ^20^, ^21^, ^22^ ^]^ In electrochemical energy storage and conversion systems, supercapacitors, metal‐ion batteries, and metal‐based batteries represent the three leading electrochemical energy‐storage technologies; and fuel cells and electrochemical water splitting systems serve as two important representatives of energy conversion technologies.^[^ ^20^, ^23^ ^]^ There is electrochemical similarity in these systems, although their working mechanisms are different. Common characters are that all devices consist of two electrodes in contact with an electrolyte layer and electrochemical interface interaction between solid electrodes and liquid electrolytes is energy‐providing process in electrochemical energy storage and conversion systems.^[^ ^20^ ^]^ In this case, as wetting behavior of solid surfaces by liquid plays a decisive role in all interfacial interaction, the wetting behavior of solid electrodes by liquid electrolytes will also decide the degree and efficiency of electrochemical electrode/electrolyte interfacial interaction and further significantly affect electrochemical energy storage and conversion performance of the electrodes. Because the configuration and the work principle of capacitive deionization are analogous to that of electrochemical energy storage and conversion systems, we also draw on wettability of the electrodes applied in capacitive deionization.

According to the reported literature, the recent research progresses of wettability control of electrode materials in electrochemical energy storage, energy conversion, and capacitive deionization could be summarized as follows: i) for supercapacitors and metal ion batteries, the better electrolyte‐wettable electrode materials generally facilitates electrolyte ion diffusion in electrode and increase ion‐accessible surface area, thereby increasing specific capacity, enhancing rate performance, and reducing interface impedance for the electrode, as well as improves energy density, power density, and cycle stability of the devices;^[^ ^24^, ^25^, ^26^, ^27^, ^28^, ^29^ ^]^ ii) in metal‐based batteries, the excellent electrolyte‐wettability of anodes could reduce nucleation overpotential, increase nucleation sites, homogenize the interfacial cation distribution, and promote the formation of thin and stable solid electrolyte interface (SEI) films, leading to the formation and growth of metal dendrites being inhibited and improving cycle stability and safety of the metal‐based batteries;^[^ ^30^, ^31^, ^32^, ^33^ ^]^ iii) the increased electrolyte‐wettability is helpful for the initial adsorption of water (reactants) on the electrode material surface and the detachment of the evolved H_2_ or O_2_ bubbles from the electrode material surface, which increases the hydrogen evolution reaction (HER) or oxygen evolution reaction (OER) catalytic kinetics of the electrode in electrochemical water splitting systems;^[^ ^34^, ^35^ ^]^ iv) similar to supercapacitors, the electrode materials used in capacitive deionization often use modification strategies of increasing electrolyte‐wettability to improve the salt adsorption capacity (SAC) and salt adsorption rate (SAR) of the electrode;^[^ ^36^ ^]^ and, v) unlike supercapacitors, metal‐ion batteries, electrochemical water splitting systems, capacitive deionization, and anodes of metal‐based batteries, the electrolyte‐philicity of electrodes are demanded moderation in gas cathodes (such as O_2_, CO_2_, and air electrodes) for metal‐based batteries and fuel cells, owing to a certain hydration state of the electrodes could ensure an adequate proton conductivity, but excessive water results in the blocked gas pathways and increases mass transport losses.^[^ ^37^, ^38^ ^]^

In view of the electrolyte‐wettability of electrodes has a remarkably impact on its electrochemical energy storage and conversion performance, the study of electrolyte‐wettability of electrode materials has spawned extensive attention across the globe. Thus, the aim of this article is to provide a comprehensive review of the topic by summarizing recent progress in the research of electrolyte‐wettability of electrode in electrochemical energy storage systems including supercapacitors, metal ion batteries, metal‐based batteries, energy conversion systems including fuel cells, electrochemical water splitting systems, and capacitive deionization (CDI), presenting critical issues, challenges, and perspectives. Such a critical and comprehensive review will guide us to deeply understand the impact mechanisms of electrolyte‐wettability of electrodes on their energy storage, energy conversion, and CDI performance, which is beneficial to improve researcher ability to design, regulate, and even control high‐performance of electrodes for electrochemical energy storage, energy conversion, and beyond.

## 2 Electrolyte‐Wettability Mechanism

### 2.1 Definition of Electrolyte‐Wettability

**Figure 1:**
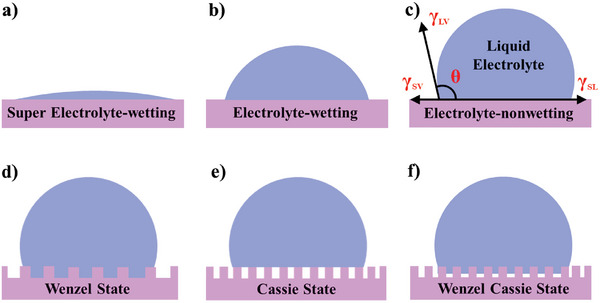
Schematic illustration of a) super electrolyte‐wetting, b) electrolyte‐wetting, and c) electrolyte‐nonwetting. Wetting model of electrode surface: d) Wenzel model, e) Cassie model, and f) Wenzel–Cassie model. Reproduced with permission.^[^ ^39^ ^]^ Copyright 2022, The Royal Society of Chemistry.

Electrolyte‐wettability could be used to embody the ability that liquid electrolytes spread out on solid electrode surface, which is usually characterized by the contact angel (CA) of the liquid electrolytes on the solid electrode surface. When a drop of electrolyte is not fully unfolded on the surface of solid electrode, the angle formed between the horizontal line of the liquid–solid interface and the tangent line of the gas–liquid interface at the rendezvous point of the gas–liquid–solid phase is called the CA (*θ*). Taking liquid electrolytes as the research object, surfaces of solid electrode could be defined as super electrolyte‐wetting (*θ* < 10°) (**Figure** **1**a), electrolyte‐wetting (10° < *θ* < 90°) (Figure 1b), and electrolyte‐nonwetting (90° < *θ* < 180°) (Figure 1c).^[^ ^4^, ^39^, ^40^, ^41^ ^]^

### 2.2 Typical Electrolyte‐Wettability Models

Yong's model. Apparent CA measurements are often necessary to well characterize the electrolyte‐wettability of electrode surface. The CAs (*θ*_Y_) on a smooth, homogeneous, rigid, insoluble, and nonreactive solid electrode surfaces are depending on surface tensions following the Young–Dupre equation^[^ ^4^, ^42^, ^43^ ^]^

(1) $$\cos\theta_{Y}=\left(\gamma_{\mathrm{SV}}--\gamma_{\mathrm{SL}}\right)/\gamma_{\mathrm{LV}}$$

where *γ*_SV_, *γ*_SL_, and *γ*_LV_ represents the solid–vapor, solid–liquid, and liquid–vapor surface tensions (Figure 1c). The electrolyte‐wettability of electrode surfaces gradually increases with the decrease of *θ*_Y_ value. However, the ideal electrode surfaces could not reach in reality.

Wenzel model. When the specific surface area of electrode is increased by increasing degree of roughness and/or macroscopic porosity, the Wenzel models are commonly employed to correlate the surface roughness with the apparent CA, and the apparent contact is described as (Figure 1d)^[^ ^4^, ^43^ ^]^

(2) $$\cos\theta_{W}=r\cos\theta_{Y}$$

where *r* defines as the ratio between the true surface area (the surface area contributed by nanopore is not considered) of electrode surface over the apparent one. It can be found that an electrolyte‐nonwettable surface (*θ*_Y_ > 90°) would become more electrolyte‐nonwettable with increase true surface area, while an electrolyte‐wettable surface (*θ*_Y_ < 90°) become more electrolyte‐wettable if the same increase of true surface area is introduced.

Cassie model. For high roughness with high true surface area, to avoid the fact that the absolute value of the right side in Equation (2) might be larger than 1, Cassie model is used to display this wetting behavior (Figure 1e)^[^ ^4^ ^]^

(3) $$\cos\theta_{C}=f_{s}\cos\theta_{s}+f_{v}\cos\theta_{v}$$

where *f*_s_ and *f*_v_ are the area fractions of the solid and vapor on the surface, respectively. Since *f*_s_ + *f*_v_ = 1, *θ*_s_ = *θ*_W_, and *θ*_v_ = 180°, Equation (3) can be written as^[^ ^4^ ^]^

(4) $$\cos\theta_{C}=-1+f_{s}\left(\cos\theta_{W}+1\right)$$

Equations (1) and (4) have sometimes been combined to a more general form to describe the CA of a rough surface^[^ ^4^ ^]^

(5) $$\cos\theta_{C}=-1+f_{s}\left(r\cos\theta_{Y}+1\right)$$

where *r* is the ratio between the true surface area over the apparent one of those parts of the solid that are in contact with liquid. From Equation (5), for high roughness with high true surface area, the change rule of electrolyte‐wettability of electrode surface with the increase of true surface area is consistent with the Wenzel models.

Wenzel–Cassie model. As shown in Figure 1f, the Wenzel–Cassie model is a transition state between the Wenzel and Cassie models.^[^ ^41^ ^]^ Liquid electrolyte droplets are semifilled on a solid electrode surface. The states transforming from the Cassie to the Wenzel model will occur in reality through electrolyte droplet extrusion, impact, or vibration.

### 2.3 Laplace Pressure

In order to more clearly elucidate the electrolyte‐wetting process on the nanoporous surface of electrodes from more microscopic perspective, an explanation based on capillary effect is adopted. When capillary tube is placed in liquid electrolyte, the liquid level in the capillary tube would become curved under the surface tensions originating from the combined action of cohesive energy of the liquid electrolyte and electrolyte‐wettable adhesion of the capillary tube surface. The capillary tube surface with high electrolyte‐wettability causes the liquid level to be concave, while the capillary tube surface with electrolyte‐nonwettability makes the liquid level convex.^[^ ^40^ ^]^ The curved liquid level in the capillary tube can give rise to additional pressure following the Young–Laplace equation

(6) $$P_{s}=F\left|\cos\theta \right|/\;\pi \;r^{2}=2\pi r\gamma \left|\cos\theta \right|/\pi r^{2}=2\gamma \left|\cos\theta \right|\;/r=2\gamma /r^{\prime }$$

where *P*_s_ represents the additional pressure, *F*cos*θ* represents the vertical resultant force (the surface tension of the curved liquid level can form a vertical resultant force, because the surface tension at every point of the interface between the curved liquid level and the capillary tube surface is tangent to the curved liquid level and they are of equal size and point to the concave side), *γ* represents the surface tension of the liquid electrolyte, *r* is the radius of the capillary tube, *r′* = *r*/|cos*θ*| is the radius of curvature of the curved liquid level, and *θ* is CA of the liquid electrolyte to the capillary tube surface.

**Figure 2:**
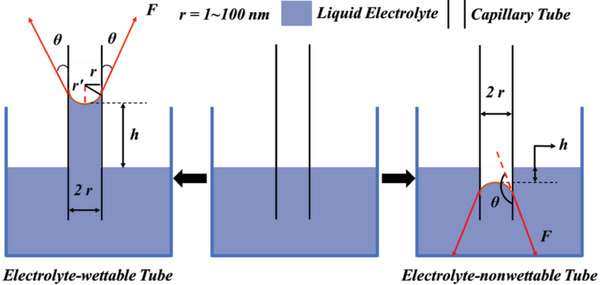
Schematic illustration of capillarity. When capillary tube is placed in the liquid electrolyte (middle), the electrolyte‐wettable capillary tube surface causes the liquid level to rise and the liquid level to be concave (left), while the electrolyte‐nonwettable capillary tube surface causes the liquid level to drop and makes it convex (right). Reproduced with permission.^[^ ^40^ ^]^ Copyright 2011, Chemical Industry Press.

As can be seen from Equation (6), when the capillary tube surface is electrolyte‐wettable (*θ* < 90°), the additional pressure *P*_s_ increases with decreasing the capillary radius and the CA, and points to the upper side of the curved liquid level and drives the curved liquid level up into the capillary tube, while the additional pressure *P*_s_ increases with decreasing the capillary radius and increasing the CA, points to the lower side of the curved liquid level and drives the curved liquid level down to flow out of the capillary tube when the capillary tube surface is electrolyte‐nonwettable (*θ* > 90°) (**Figure** **2**).^[^ ^40^ ^]^ The additional pressure that drives the liquid electrolyte into the capillary tube is commonly referred to as the capillary forces. Thus, the more electrolyte‐wettable capillary tube surface is, the easier it is for the electrolyte to access the capillary tube and transmit in the capillary tube, and the dependence of the accessibility and transmission of electrolyte on the electrolyte‐wettability increases with the decrease of the capillary tube radius. By contrast, the more electrolyte‐nonwettable the capillary tube surface is, the more difficult it is for the electrolyte to access the capillary tube and transmit in the capillary tube, and the difficulty of access and transmission of the electrolyte becomes greater as the decrease of the capillary tube radius. If the nanopores of electrode are regarded as the capillary, the above analysis of Laplace pressure clearly reveals the influence mechanism of electrolyte‐wettability on electrochemical utilization of the nanopore surfaces.

### 2.4 Surface Energy and Surface Tension

Surface energy is the embodiment of intermolecular force on the surface of an object, which is closely related to surface wettability. The surface energy of solid electrode is usually calculated by the CA, the surface tension of liquid electrolytes, and the interfacial tension between the solid electrode and liquid electrolytes. The surface tension of liquid electrolytes is a manifestation of solvent molecular force that occurs at the boundary of the liquid electrolytes and gas contact.^[^ ^39^ ^]^ Although surface energy is energy and surface tension are force, and the two represent different physical meanings, they are numerically and dimensionally the same.^[^ ^44^ ^]^ Thus, according to Young–Dupre equation, high surface energy of electrode, low interfacial energy of the electrode with liquid electrolytes, and low surface energy of the liquid electrolytes are benefit for the liquid electrolytes wetting the electrode.

### 2.5 Measures to Improve Electrolyte‐Wettability of Electrode Materials

Based on Young–Dupre equation, there are three measures to improve electrolyte‐wettability of electrode materials from the perspective of thermodynamics.

Increasing surface energy of electrode. The surface energy of electrode materials could be increased by enhancing amorphous region,^[^ ^45^ ^]^ constructing more defect sites,^[^ ^46^, ^47^ ^]^ and introducing ultrafine nanostructure in the electrode material surface.^[^ ^48^, ^49^ ^]^

Decreasing interfacial energy of electrode materials with liquid electrolytes. Liquid electrolytes mainly contain electrolyte ions and solvent. Therefore, on the one hand, the interfacial energy of electrode with liquid electrolytes could be decreased by enhancing the adsorption energy^[^ ^50^, ^51^ ^]^ and binding energy,^[^ ^52^ ^]^ constructing the similar polarity (according to the principle of similar polarity compatibility),^[^ ^53^ ^]^ and forming hydrogen bond^[^ ^54^, ^55^ ^]^ between the electrode materials surface and the solvent from the electrolytes. On the other hand, increasing the adsorption energy,^[^ ^56^ ^]^ binding energy,^[^ ^57^ ^]^ and electrostatic interaction^[^ ^58^ ^]^ between the electrolyte ions and the electrode material surface (these interactions are generally referred to collectively as ion‐philicity) could also decrease the interfacial energy.

Decreasing surface energy of liquid electrolytes. The decremental of surface energy of liquid electrolytes could be accomplished by the additives that have low surface energy and are able to reduce intermolecular forces of the electrolyte solvent.^[^ ^59^, ^60^ ^]^

## 3 Electrolyte‐Wettability of Electrode Materials in Electrochemical Energy Storage Systems

In electrochemical energy storage systems including supercapacitors, metal ion batteries, and metal‐based batteries, the essence that electrodes store energy is the interaction between electrode active materials and electrolyte ions, which is significantly affected by the contact state of the electrode active material surface with electrolyte ions.^[^ ^61^ ^]^ However, it is well known that the electrolyte ions are difficult to independently contact the surface of electrode active materials, because the electrolyte ions usually dissociated in electrolyte solvents at room temperature and the electrolyte ions have higher diffusion and mobility in liquid solvent environment compared to those in gas or vacuum.^[^ ^62^ ^]^ Therefore, the mutual wettable interface between electrode active materials and liquid electrolytes ensures sufficient contact of the electrode active material surface with the electrolyte ion from liquid electrolytes, thereby optimizing electrochemical energy storage performance of the electrode prepared from the electrode active materials. In addition, the wettability of electrode active materials with liquid electrolytes could also be improved by enhancing the wettability of other components of the electrode, such as substrate, coating layer, and current collectors, since the electrode active materials is often dispersed in them or loaded on them. **Table** **1** presents some electrolyte‐wettable electrodes materials used in various energy storage systems.

**Table 1:** A summary of electrolyte‐wettable materials and their applications in energy storage systems

Materials	Component	Electrode	Electrolyte	Wettability/CA [*θ*]	Application	Refs.
*N*‐doped macroporous carbon film	Active materials	Cathode/anode	1 m H_2_SO_4_	74°	Supercapacitors	[63]
*N*‐doped ordered mesoporous few‐layer carbon	Active materials	Cathode/anode	0.5 m H_2_SO_4_	21°	Supercapacitors	[24]
Ordered mesoporous carbons (OMCs) grafted with poly(*N*‐vinylpyrrolidone)	Active materials	Cathode/anode	2 m KOH	36 .8°	Supercapacitors	[25]
N, S, O codoped microporous carbon nanosheets	Active materials	Cathode/anode	6 m KOH	83.6°	Supercapacitors	[64]
Hierarchical nitrogen‐doped carbon nanocages	Active materials	Cathode/anode	6 m KOH	48.3°	Supercapacitors	[53]
B, N, and O codoped superhydrophilic porous carbon	Active materials	Cathode/anode	1 m H_2_SO_4_	63°	Supercapacitors	[65]
Electrolyte‐philic polymer grafted interconnected hierarchically porous carbon	Active materials	Anode	2 m KOH	55.7°	Supercapacitors	[66]
Block carbon electrode with vertical channel array	Active materials	Cathode/anode	6 m KOH	0°	Supercapacitors	[67]
Cellulose nanofibrils/carbon nanotubes/reduced graphene oxide	Active materials	Cathode/anode	1 m H_2_SO_4_	0°	Supercapacitors	[68]
Fish scale‐like PANI nanorod‐decorated carbon nanofiber/nanosheet composite	Active materials	Cathode	1 m H_2_SO_4_	22.4°	Supercapacitors	[69]
Cl^−^ modified polyethersulfone	Substrates	Anode	6 m KOH	62.4°	Supercapacitors	[70]
Vermiculite‐coated CoB	Active materials	Cathode	6 m KOH	17.2°	Supercapacitors	[71]
Nanostructure PANI	Active materials	Cathode	1 m H_2_SO_4_	29°	Supercapacitors	[45]
N‐doped 3D carbon network confined nanosized SnSb alloy	Active materials	Anode	0.5 m KPF_6_ in DME	15.5°	K^+^ batteries	[48]
N, P codoped carbon nanofibers decorated with MoP ultrafine nanoparticles	Active materials	Anode	0.8 m KPF_6_ in EC and diethyl carbonate (DEC) (V/V, 1:1)	0°	K^+^ batteries	[72]
Aryl‐trifluoromethanesulfonylimide moiety grafted on carbon layer	Substrates	Cathode	1 m LiPF_6_ in EC/DEC/DMC (V/V/V, 1:1:1)	76°	Li^+^ batteries	[73]
Mg_1.5_VCr(PO_4_)_3_	Active materials	Cathode	2 m NaClO_4_ in acetonitrile and water (V/V, 4.1:0.9)	14°	Mg^2+^ batteries	[74]
F‐free Ti_3_C_2_/S composite	Active materials	Cathode	1 m LiTFSI with 1 wt% LiNO_3_ in DOL and DME (V/V, 1:1)	19.4°	Li–S batteries	[75]
PDA‐coated carbon nanotubes	Substrates	Cathode	1 m LiTFSI in tetra(ethylene glycol)dimethyl ether	42.3°	Li‐air batteries	[76]
Li–Al–O layer coated 3D Al_2_O_3_	Current collector	Anode	1 m LiPF_6_ in EC and DEC (V/V, 1:1)	Almost 0°	Li metal batteries	[77]
Poly(vinyl butyral)‐coated Zn	Active materials	Anode	1 m ZnSO_4_	72.2°	Zn metal batteries	[78]
Poly(vinylidene difluoride) coated Zn	Active materials	Anode	3 m ZnSO_4_	16°	Zn metal batteries	[79]
Silk fibroin coated Zn	Active materials	Anode	2 m ZnSO_4_	15.55°	Zn metal batteries	[80]
Polypyrrole coated Zn	Active materials	Anode	2 m ZnSO_4_	32.75°	Zn metal batteries	[81]
(3‐aminopropyl)triethoxysilane coated Zn	Active materials	Anode	2 m ZnSO_4_	13.9°	Zn metal batteries	[82]
Cu–Zn alloy coated Zn	Active materials	Anode	2 m Zn(CF_3_SO_3_)_2_	66.8°	Zn metal batteries	[83]
Zinc oxalate coated Zn	Active materials	Anode	2 m ZnSO_4_	15.86°	Zn metal batteries	[55]
ZnSe‐coated Zn	Active materials	Anode	2 m ZnSO_4_	85.6°	Zn metal batteries	[84]
Amorphous SiN coated Zn	Active materials	Anode	2 m ZnSO_4_	28.4°	Zn metal batteries	[85]
Dual‐channel 3D porous Zn	Active materials	Anode	2 m Zn(CF_3_SO_3_)_2_	42°	Zn metal batteries	[86]

### 3.1 Electrolyte‐Wettability in Supercapacitors

**Figure 3:**
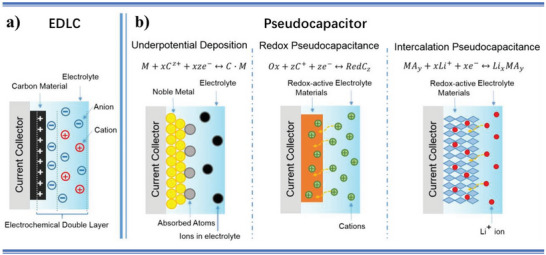
Schematics of charge‐storage mechanisms for a) an EDLC and b) different types of pseudocapacitor (underpotential deposition pseudocapacitor, redox pseudocapacitor, and ion intercalation pseudocapacitor from left to right). (a) Reproduced with permission.^[^ ^19^ ^]^ Copyright 2018, American Chemical Society. (b) Reproduced with permission.^[^ ^88^ ^]^ Copyright 2014, The Royal Society of Chemistry.

For the decades, supercapacitors have been extensively studied in electrochemical energy storage due to their ultrahigh power density, good stability, and long cycle life.^[^ ^87^ ^]^ Supercapacitors store charges at the interface of electrode/electrolyte, via electrostatic adsorption of electrolyte ions onto the charged surface of electrode, for electrochemical double‐layer capacitors (EDLCs) (**Figure** **3**a); and through fast and nondiffusion limited faradaic redox reactions on electrode/electrolyte interface for pseudocapacitors (Figure 3b).^[^ ^19^, ^88^, ^89^ ^]^ It is generally acknowledged that carbon‐based electrode active materials mainly exhibit double‐layer energy storage behavior and metallic compound and conducting polymer electrode active materials principally express pseudocapacitive energy storage behavior.^[^ ^19^, ^90^ ^]^ However, the fact that the electrode/electrolyte interface interaction is essence of electrochemical energy storage for supercapacitors is not affected by the electrochemical energy storage types and species of electrode active materials. The electrolyte‐wettability of electrodes play a key role in electrode/electrolyte interface interaction of high efficiency and further in electrochemical energy storage performance of supercapacitors, which is attributed to the good electrolyte‐wettability increasing ion‐accessible surface area of electrode, facilitating ion diffusion in the electrode channel, and promoting ion movement on the electrode surface.^[^ ^53^ ^]^ In supercapacitors, improving electrolyte‐wettability of electrodes mainly includes improving electrolyte‐wettability of electrode active materials and substrates of the electrodes.

#### 3.1.1 Carbon‐Based Electrode Active Materials for Supercapacitors

##### Toward Aqueous Electrolyte

In generally, carbon materials with nonpolar surfaces are difficult to be wetted by strongly polar aqueous solutions, preventing water from getting into small pores, thus limits the effective surface area of carbon materials as electrode active materials.^[^ ^53^ ^]^ Hence, improving electrolyte‐wettability of carbon materials is a valid solution to maximize the supercapacitive potential of carbon electrode materials in aqueous electrolyte.

**Figure 4:**
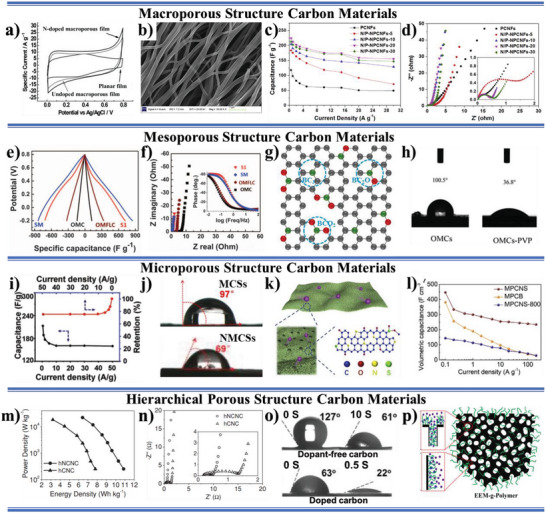
a) Supercapacitor performance of macroporous carbon film. N‐doped macroporous carbon film showing high specific capacitance owing to a large surface area and superior electrolyte‐wettability. Reproduced with permission.^[^ ^63^ ^]^ Copyright 2010, Wiley‐VCH. b) SEM image of N/P‐NPCNFs‐20 (where the numbers (5, 10, 20, and 30) represent the mass ratio of C and N source to P source in the electrospinning solution), c) specific capacitance of each sample at different current densities (0.5–30 A g^−1^), and d) Nyquist plots of all samples in the frequency range of 100 kHz and 10 mHz (the inset is the magnified region). Reproduced with permission.^[^ ^26^ ^]^ Copyright 2014, Elsevier. e) Galvanostatic charging/discharging test from the first cycle at 1 A g^−1^ and f) complex‐plane plots of AC impedance for OMC, OMFLC, OMFLC‐S1, and OMFLC‐SM) (the inset shows phase angle versus frequency). Reproduced with permission.^[^ ^24^ ^]^ Copyright 2015, American Association for the Advancement of Science. g) A schematic illustration of B‐containing groups in the BKB carbon structure (black = carbon, green = boron, and red = oxygen). Reproduced with permission.^[^ ^91^ ^]^ Copyright 2022, Elsevier. h) Water CA test of OMCs and OMCs‐PVP samples. Reproduced with permission.^[^ ^25^ ^]^ Copyright 2016, Elsevier. i) Specific capacitance and its retention ratio at different current densities for NMCSs and j) water CA test of NMCSs and MCSs samples (undoped microporous carbon sheets). Reproduced with permission.^[^ ^92^ ^]^ Copyright 2014, Wiley‐VCH. k) The mechanism of energy storage and possible atomic configuration for O, N, and S incorporation in MPCNS electrode materials, and l) volumetric capacitance of MPCNS, MPCB, and MPCNS‐800 electrode at the current densities from 0.1 to 200 A g^−1^. Reproduced with permission.^[^ ^64^ ^]^ Copyright 2018, Elsevier. m) Ragone plots and n) Nyquist plots of the hNCNC‐SC and hCNC‐SC in 6 m KOH electrolyte (the inset magnifies the high‐frequency range). Reproduced with permission.^[^ ^53^ ^]^ Copyright 2015, Wiley‐VCH. o) Dynamic water CA measurement results of dopant‐free carbon and doped carbon. Reproduced with permission.^[^ ^65^ ^]^ Copyright 2021, The Royal Society of Chemistry. p) Schematic illustration of EEM‐*g*‐polymer. Reproduced with permission.^[^ ^66^ ^]^ Copyright 2019, American Chemical Society.

Macroporous carbon electrode materials. The macropores in macroporous carbon electrode materials more mainly allow an efficient transport route for the electrolyte ion and its core space can be used as a buffering reservoir of electrolyte to minimize the diffusion distance of electrolyte than increase limited specific surface area.^[^ ^93^, ^94^ ^]^ However, macroporous carbon electrode materials with inferior electrolyte‐wettability do not allows electrolyte to absolutely contact with surface of those macropore, leading to unsatisfactory supercapacitive performance. A macroporous carbon film is doped by post‐treatment approach under N‐containing NH_3_ precursors.^[^ ^63^ ^]^ The doped macroporous carbon film is determined by XPS to contain 11% N, which confirms that N is successfully doped into the sample. Because N atoms incorporating could increase polarity,^[^ ^95^ ^]^ surface energy,^[^ ^46^ ^]^ and binding energy with electrolyte ions^[^ ^96^ ^]^ of the macroporous carbon film, the N‐doped macroporous carbon film exhibits better electrolyte‐wettability in aqueous electrolyte than the undoped samples.^[^ ^63^ ^]^ Thus, the N‐doped macroporous carbon film as electrode materials exhibits a rectangular shape at a high scan rate of 100 mV s^−1^ over a potential range of 0–0.8 V in 1 m H_2_SO_4_ aqueous solution, which is consistent with good charge propagation at the electrodes materials. The specific capacitances of the N‐doped macroporous carbon film electrode materials are measured to be 103.2 F g ^−1^ in 1 m H_2_SO_4_ aqueous solution (**Figure** **4**a). By contrast, the capacitance from an undoped macroporous carbon film shows only 86.7 F g ^−1^. The enhancement of specific capacitance is attributed to the improved electrolyte‐wettability by N‐doping. Although there are no pores in the fibers for nonporous carbon nanofibers (NPCNFs), the gap between nanofibers could form interstitial pore in macroporous size range (Figure 4b). The N, P codoped NPCNFs (N/P‐NPCNFs) exhibit superior supercapacitive performance, because the N, P codoping enhances the electrolyte‐wettability of the electrodes, which facilitates the formation of electrochemical double‐layer (EDL).^[^ ^26^ ^]^ When P content increases to 9.01%, the electrode active materials could obtain high specific capacitance of 224.9 F g^−1^ at 0.5 A g^−1^ in 1 m H_2_SO_4_, which maintains high capacitance retention ratio of 70% at 30 A g^−1^ (Figure 4c), small *R*_ct_ (Figure 4d), low equivalent series resistance (ESR), and robust long‐term stability. Furthermore, N/P‐NPCNFs‐20 in a two‐electrode device also demonstrates high specific capacitance of 168 F g^−1^ at 0.5 A g^−1^ and good rate capability with a high capacitance retention ratio of ≈77.0% at 15 A g^−1^.

Mesoporous carbon electrode materials. The most attractive property of mesopores in mesoporous active materials is to transport electrolyte ion and to provide higher specific surface area. Unfortunately, inferior electrolyte‐wettability makes it possible for a small number of electrolyte ions to entry those mesopores, resulting in a decrease in transport efficiency and specific surface area utilization of those mesopores. The mixture of N‐doped ordered mesoporous few‐layer carbon (OMFLC‐N‐SM, the mixture at the ratio of OMFLC‐N‐S1:OMFLC‐N‐S2:OMFLC‐N‐S3 = 0.3:0.3:0.4; OMFLC‐N‐S1, OMFLC‐N‐S2, and OMFLC‐N‐S3 represent N‐doped ordered mesoporous few‐layer carbon (OMFLC‐N) with 8.2% N contents, OMFLC‐N with 7.5%, and OMFLC‐N with 11.9%, respectively.) with improving electrolyte‐wettability has a specific capacitance as high as 855 F g^−1^ at a current density of 1 A g^−1^ in aqueous electrolytes^[^ ^24^ ^]^ (Figure 4e). Over a wide range of current densities, The OMFLC‐N‐SM continued to provide a well‐behaving galvanostatic charging and discharging curve and high rate performance, achieving 615 F g^−1^ at 40 A g^−1^. Electrochemical impedance spectroscopy (EIS) is used to find the lowest ESR of ≈0.8 Ω for OMFLC‐N‐S1, better than those of ordered mesoporous few‐layer carbon (OMFLC) and ordered mesoporous carbon (OMC) (Figure 4f). This may be attributed to better wetting on OMFLC‐N, which lowers the interfacial resistance. The >45° (negative) phase angle of both OMFLC‐N (S1 and SM; inset of Figure 4f) at relatively high frequencies confirms their capacitive behavior at fast rates. After the mesoporous Ketjen black (KB) carbon is doped with boron (B) atoms, the CA of the B‐doped KB (BKB) with rich B‐containing groups (Figure 4g) toward KOH electrolyte is 75°, which is much lower than that of the KB (114°), and the same trend is observed with H_2_SO_4_ electrolyte (119° and 138°, respectively).^[^ ^91^ ^]^ The BKB as electrode active material of supercapacitors exhibit a high areal capacitance of 1.67 mF cm^−2^ and a low ESR of 0.085 Ω. Some polar functional groups have very similar polarity to aqueous solution and can be easily wetted by aqueous electrolytes based on similar polarity compatibility. Thus, an approach of introducing polar functional groups on the surface of the ordered 3D cubic mesoporous carbon (CMK‐8) is carried out by nitric acid treatment to promote the electrolyte‐wettability of the CMK‐8 in aqueous electrolyte.^[^ ^97^ ^]^ The acid‐modified CMK‐8 exhibits the largest specific capacitance of 246 F g^−1^ at a current density of 0.625 A g^−1^ in 2 m KOH electrolyte and the lower impedance on electrode/electrolyte interface. The grafting technique that hydrophilic poly(N‐vinylpyrrolidone) (PVP) brushes are introduced on the surface of OMC by SI‐eATRP results in the improvement of electrolyte‐wettability (Figure 4h) and a remarkable increase in the specific capacitance.^[^ ^25^ ^]^ The specific capacitances of the ordered mesoporous carbons grafted with hydrophilic PVP (OMCs‐PVP) are 253 F g^−1^ at 0.5 A g^−1^, which is more than twice that of the OMCs electrode (92 F g^−1^ at 0.5 A g^−1^). When the charging and discharging current increases from 0.5 to 5 A g^−1^, the capacitance retention ratios are 64% and 81% for OMCs and OMCs‐PVP, respectively, indicating that this OMCs‐PVP exhibits a high‐power density. These results are attributed to the hydrophilic nature and the adsorption to electrolyte ions of the OMCs‐PVP electrode materials.

Microporous carbon electrode materials. Microporous carbon‐based electrode materials possess high theoretical specific area under maintaining low pore volume to prevent screening effect, which supplies high EDLC.^[^ ^98^ ^]^ However, the electrolyte‐nonwettable surfaces lead to poor diffusion kinetics of electrolyte ions and low efficient surface area for charge storage in aqueous electrolytes. 2D N‐doped microporous carbon sheets (NMCSs) by direct heat treatment under N‐containing ionic liquid precursors with electrolyte‐wettable surface show high specific capacitance, excellent rate capability, and low ion‐transport resistance in aqueous electrolyte.^[^ ^92^ ^]^ The specific capacitance of the NMCSs is 213 and 160 F g^−1^ at 0.5 and 10 A g^−1^, respectively, and the specific capacitance of 160 F g^−1^ is maintained over a wide range of current density from 10 to 50 A g^−1^ (Figure 4i). The good electrolyte‐wettability (Figure 4j) originating from the N‐doped polar surface is crucial site for strengthening the capacitance and power property and reducing ion‐transport resistance in the solid–liquid interface. Analogous to NMCSs, the prepared N, S, O codoped microporous carbon nanosheets (MPCNS) (Figure 4k) have also enhancing electrolyte‐wettability and excellent capacitive performance.^[^ ^64^ ^]^ Compared the porous carbon nanosheet heat‐treated at 800 °C (MPCNS‐800) only showing a low volumetric capacitance of 144 F cm^−3^ and the volumetric capacitance of 382 F cm^−3^ for the microporous carbon electrode materials prepared by conventional KOH activation (MPCB), the MPCNS electrode materials with good electrolyte‐wettability could exhibit volumetric capacitance of 447 F cm^−3^ at the current density of 0.1 A g^−1^ (Figure 4l). Furthermore, the MPCNS electrode materials still exhibit a high volumetric capacitance of 300 F cm^−3^ at the current density of 1 A g^−1^, which maintains 235 F cm^−3^ even at the ultrahigh current density of 200 A g^−1^ (Figure 4l). The two‐electrode system is further evaluated using the assembled MPCNS||MPCNS symmetric supercapacitors. A high volumetric energy density of 12.5 Wh L^−1^ could be obtained at the volumetric power density of 24 W L^−1^, which maintains 3.1 Wh L^−1^ even at the volumetric power density of 9.67 kW L^−1^.

Hierarchical porous carbon electrode materials. The hierarchical porous carbon electrode materials, having interconnected macropores, mesopores, and micropores, are expected to have shorter transport distance, richer transport channels, and higher accessible specific surface area of electrolyte. However, the poor electrolyte‐wettability of the hierarchical porous carbon materials causes that the advantages of hierarchical porosity are hard to play, including rapid ion‐transport ways and more efficient surface area for charge storage.^[^ ^94^ ^]^ The hierarchical N‐doped carbon nanocages (hNCNCs) incorporating rich N atoms feature a large specific surface area, multiscale porous structure, and much improved electrolyte‐wettability.^[^ ^53^ ^]^ Figure 4m shows the typical electrochemical performances for the hNCNC and hierarchical carbon nanocages (hCNCs) based supercapacitors (hNCNC‐SC and hCNC‐SC). The hNCNC electrode materials give higher gravimetric capacitance of 313 F g^−1^ at 1 A g^−1^ and 234 F g^−1^ at 100 A g^−1^ (≈75% retention) and the hNCNC‐SC reaches higher energy density of 10.90 and 6.42 Wh kg^−1^ with the corresponding average power density of 0.25 and 22.22 kW kg^−1^ than the hCNC electrode materials and the hNCNC‐SC, respectively. To further ascertain the effect of electrolyte‐wettability on electrochemical energy storage performance, the electrochemical impedance spectroscopy is performed as shown in Figure 4n. By extrapolating the vertical portion to the real axis, the difference of 1.08 Ω for the two ESR values mainly results from the smaller *R*_ct_ of the hNCNC‐SC (0.23 Ω) than the hCNC‐SC (1.32 Ω), with 1.09 Ω difference (inset in Figure 4n). The characteristic frequency ƒ_0_ at the phase angle of −45° is 2.58 Hz for the hNCNC‐SC and 1.01 Hz for the hCNC‐SC, corresponding to the time constant *τ*_0_ (*τ*_0_ = 1/*ƒ*_0_) of 0.39 and 0.99 s, indicating the faster charging and discharging rate and the higher power density for the former thereof. The much smaller *R*_ct_ and the much shorter time constant for the hNCNC‐SC than the hCNC‐SC originates from the larger ion‐accessible surface area and the faster ion diffusion due to the much better electrolyte‐wettability for the former. Biomass is widely considered to be a promising precursor of polar atom doped hierarchical porous carbon due to its renewability, structural diversity, and abundant heteroatoms. Shen and co‐workers prepare a N, S codoped interconnected hierarchical porous carbon with electrolyte‐wettability using green radish as precursor.^[^ ^99^ ^]^ An innovative closed‐loop and scalable process to produce B, N, and O codoped superhydrophilic porous carbon derived from waste sweet potato leaves is proposed by Qiu's group.^[^ ^65^ ^]^ The B, N, and O polar atoms that are in situ introduced into the skeleton of carbon significantly improve the surface wettability toward aqueous electrolyte (Figure 4o), which greatly contributes to the excellent electrochemical performance for supercapacitor applications. Our research group grafts various electrolyte‐philic polymer brushes onto the interconnected hierarchically porous carbon (IHPC) electrode active material surface for ameliorating its electrolyte‐wettability and increasing the utilization efficiency of porous structure (Figure 4p).^[^ ^66^ ^]^ The electrochemical energy storage performance of IHPC and electrolyte‐philic electrode materials (EEM)‐*g*‐polymer in a three‐electrode system using 2 m KOH aqueous electrolyte is investigated. The area enclosed by the CV curves of EEM‐*g*‐polymer increases profoundly compares with that of IHPC, indicating that specific capacitance is obviously improved after grafting the polymer brushes, likely due to the enhanced electrolyte‐wettability. The calculated specific capacitances of pristine IHPC, EEM‐*g*‐PAN, EEM‐*g*‐PAM, EEM‐*g*‐PVP, and EEM‐*g*‐PSSNa are 75, 180, 220, 254, and 251 F g^−1^, respectively. The specific capacitances of EEM‐*g*‐PAM, EEM‐*g*‐PVP, and EEM‐*g*‐PSSNa electrode materials are ≈3 times higher than that of pristine IHPC. The rate performance of IHPC pristine and EEM‐*g*‐polymer electrode materials demonstrates that the specific capacitance remains ≈73% of initial specific capacitance when the current density increased from 1.0 to 5.0 A g^−1^. All EEM‐*g*‐polymer samples except EEM‐*g*‐PAN exhibits smaller *R*_s_ than that of IHPC pristine in the EIS curves, indicating hydrophilic polymer brushes reduce the contact resistances between electrode material and electrolyte and the hydrophobic polymer brushes like ‐PAN polymer increase the contact resistances. Furthermore, it should be noted that EEM‐*g*‐polymer electrodes display larger *R*_ct_ and *R*_w_ values than IHPC electrode, possibly due to the negative contribution of polymer brushes to migration and adsorption of electrolyte ions resulting from the steric hindrance of the polymer brushes.

**Figure 5:**
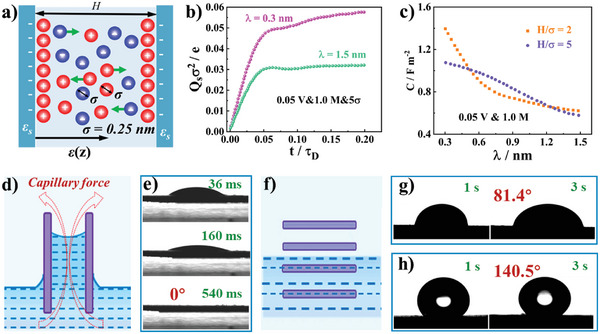
a) Schematic diagrams of representative slit pore from the porous carbon materials. b) Change of the surface charge density with time under different conditions (*λ* = 0.3 nm represents a solvent‐philic surface and *λ* = 1.5 nm represents a solvent‐phobic surface). c) Capacitance against the surface wetting parameter under *H*/*σ* = 2 and *H*/*σ* = 5. Reproduced with permission.^[^ ^100^ ^]^ Copyright 2022, American Chemical Society. d) Capillary effect diagram. e) CA of WCS, f) noncapillary effect diagram, CA of g) longitudinally cut block carbon materials activated at 900 °C, and h) conventional powder carbon materials. Reproduced with permission.^[^ ^67^ ^]^ Copyright 2021, Elsevier.

Comparing the effect of electrolyte‐wettability on supercapacitive performance of carbon electrode materials with various structures, according to the above analysis and evaluation, it is found that improving electrolyte‐wettability of microporous carbon, mesoporous carbon, microporous carbon, and hierarchical carbon is conducive to the increase of their respective supercapacitive performance. However, it is not clear which porous carbon materials are most affected by electrolyte‐wettability. Therefore, the effect of electrolyte‐wettability on the supercapacitive performance of carbon electrode materials is revealed by static and dynamic density functional theory.^[^ ^100^ ^]^ First, the model of pore in carbon electrode materials is assumed as shown in **Figure** **5**a. To describe the charging process, the surface charge density, *Q*_s_ could be obtained by applying the charge neutrality condition when^[^ ^101^ ^]^

(7) $$Q_{s}\left(t\right)=-\int_{0}^{H/2}\sum_{i}eZ_{i}\rho_{i}\left(z,t\right)dz$$

At thermodynamic equilibrium, the surface charge density reaches a constant value, designated as $Q_{s}^{e}$, and then the integral capacitance could be determined by

(8) $$C=Q_{s}^{e}/V_{s}$$

where *H* is pore width, e is the elementary, *Z_i_* is the valence of ion species (*i* = +, −), *ρ_i_* (*z*, *t*) is ion local density, *z* is the distance to the left charged surface, *t* is the charging duration time, and *V*_s_ is the applied surface voltage. The surface charge density is calculated through Equation (7), and the time‐dependent ionic density in the like‐charged pore is calculated with dynamic density functional theory (Figure 5b). It is found that electrolyte‐wettable pore surface has higher plateau value of the surface charge density than that of the electrolyte‐nonwettable pore surface. According to Equation (8), the capacities of pores with various electrolyte‐wettable surfaces are calculated. Whether the pore width is *H*/*σ* = 2 or 5 (*σ* is the ion size), the capacity decreases with the increase of *λ* (*λ* is the microscopic parameter to scale electrolyte‐wettability of the surface, and the smaller the value of *λ* is, the more electrolyte‐wettable the pore surface is^[^ ^102^ ^]^), showing that the electrolyte‐wettable surface is beneficial to improving the capacity of the pore in electrode materials, as well as the smaller the pore width is, the more obvious the beneficial effect is (Figure 5c).^[^ ^100^ ^]^ Our group conduct experiments to compare the ability of electrolyte‐wettability to improve the supercapacitive performance of macroporous, mesoporous, and hierarchical porous carbon materials and find the ranking of improvement degree at the same electrolyte‐wettability: hierarchical porous carbon > mesoporous carbon > macroporous carbon.^[^ ^103^, ^104^ ^]^ Our experimental results are basically in agreement with the simulation results. Moreover, a new type of block carbon electrode with vertical channel array (WCS) is prepared by carbonizing the wood block with natural conduit/sieve structure.^[^ ^67^ ^]^ Thanks to capillary effects caused by vertical channel array (Figure 5d), the CA of WCS is 0° after 540 ms (Figure 5e), which is significantly less than that of longitudinally cut block carbon materials (81.4° after 3 s) and conventional powder porous carbon materials (140.5° after 3 s) without capillary effect (Figure 5f–h), under basically the same composition and crystal structure of all block carbon materials. An obvious conclusion that the vertical channels array is the key point to improve the electrolyte‐wettability of block carbon materials could be drawn. The novel electrolyte‐wettable carbon electrode enjoys satisfactory specific capacitance of 2452 mF cm^−2^ at 5 mA cm^−2^.

Carbon‐based composite electrode materials. Carbon‐based composite electrode active materials would be able to combine the physical and chemical strengths of each part of the composites for supercapacitors. Nevertheless, the capacitive performance of the composites is also limited by the inferior electrolyte‐wettability of carbon materials part and/or noncarbon part. N atoms doped in the carbon bulk^[^ ^105^ ^]^ and/or introducing O‐containing functional groups (including C=O and N—O) on the surface of the carbon bulk^[^ ^106^ ^]^ would enhance the electrolyte‐wettability of the VN/C composite electrode, enlarge contact area between the electrode and electrolyte, and facilitate the migration of electrolyte ions, which would further enhance the capacitive performance of the VN/C composite material. In addition to the above methods, electrostatic adsorption is also used to graft hydrophilic poly(allylamine hydrochloride) (PAH) on the surface of VN/C composite electrode materials to improve the electrolyte‐wettability in aqueous electrolyte.^[^ ^107^ ^]^ This method is simple and mild to operate and may not destroy the structure of the electrode active material. The capacitive performance of VN/C‐PAH and PAH‐free control materials is evaluated. Compared to PAH‐free control electrode materials, the VN/C‐PAH electrode materials yield higher specific capacitance of 96.4 F g^−1^ at the current density of 0.5 A g^−1^, while the rate performance remains ≈57% (5 to 0.5 A g^−1^). The reason is that the hydrophilic PAH chains could increase the contact area between the electrode materials and electrolyte, and enhance the available effective specific surface area of the composite electrode material during charging and discharging, thus promoting the adsorption of electrolyte ions during the charging process and improving the electrochemical energy storage performance of the composite electrode material. The biomimetic fish scale‐like polyaniline (PANI) not only provides pseudocapacitance but also probably enhances the electrolyte spreading capacity in the electrode/electrolyte interfaces under the strong capillary forces.^[^ ^69^ ^]^ Thus, a fish scale‐like PANI nanorod‐decorated carbon nanofiber/nanosheet composite prepared by in situ polymerization of PANI on the surface of an interwoven carbon nanofiber/nanosheet network presents favorable electrolyte‐wettability. The supercapacitor device assembled by the electrolyte‐wettable composite exhibits a great energy density of 65.3 Wh kg^−1^ and large power density of 8 kW kg^−1^.

The substrate of carbon electrode materials. To enhance the flexibility of electrode for adapting the demand of smart and wearable electronics, the film electrodes are fabricated using polyethersulfone (PES) as a substrate polymer, and activated carbon as an active substance.^[^ ^108^ ^]^ However, with a view to hydrophobicity of PES, the film electrodes are modified by introducing hydrophilic halogen functional groups^[^ ^70^ ^]^ or grafting amphiphilic poly(acrylic acid)(PAA)‐*b*‐poly(acrylonitrile)(PAN)‐*b*‐PAA^[^ ^109^ ^]^ to enhance the electrolyte‐wettability of the film electrodes. The principle that grafting amphiphilic copolymers could improve film electrodes is that the hydrophilic block ‐PAA of the amphiphilic copolymer is induced to direct on the surface of the film electrode for being wetted by aqueous electrolyte, whereas the hydrophobic block of ‐PAN is embedded in the PES substrate during phase‐separation process. The modified films electrode shows high specific capacitance of 310 F g^−1^ at 0.5 A g^−1^ in the alkaline electrolyte using a three‐electrode system and the symmetric device using the modified films as two electrodes and using 1 m Na_2_SO_4_ aqueous solution as an electrolyte yields a high energy density of 17.8 W h kg^−1^ at a power density of 160 W kg^−1^.

##### Toward Organic Electrolyte

**Figure 6:**
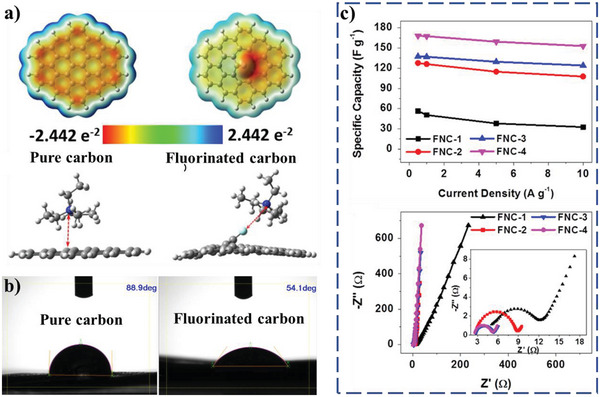
a) The simulated electrostatic potential surface and optimized configuration of TEA^+^ adsorbed for pure carbon and fluorinated carbon. b) CA of electrolyte (TEABF_4_) on pure carbon and fluorinated carbon and c) relationship of the specific capacitance and current densities and Nyquist plots of different fluorinated carbons electrode materials (F contents of FNC‐1, FNC‐2, FNC‐3, and FNC‐4 are 7.8%, 8.1%, 12.9%, and 17.5%, respectively). Reproduced with permission.^[^ ^110^ ^]^ Copyright 2016, Elsevier.

Commercial EDLCs generally use quaternary ammonium salt dissolving in acrylonitrile (AN) and/or polycarbonate (PC) as organic electrolyte, because of high operating voltage, wide working temperature, and stable chemical performance. It is well known that pure carbon materials are nonpolar and organic electrolyte is slight polar. Thus, it is generally assumed that the surface of carbon electrode materials can be well wetted by organic electrolyte, leading to rare report regarding improving electrolyte‐wettability of carbon electrode materials toward organic electrolyte. However, the nonpolar surface of carbon electrode materials is not conducive to the adsorption and movement of electrolyte ions during charging and discharging process. Beside reducing the amount of polar O atoms of carbon electrode active materials would exhibit better electrolyte‐wettability toward organic electrolytes and electrochemical energy storage performance,^[^ ^27^ ^]^ fluorinated carbon electrode active materials (FNCs) with rich semi‐ionic C—F bonds also show a stronger ion‐philicity and even electrolyte‐wettability in organic electrolyte (**Figure** **6**a), which facilitates transport of electrolyte ions within the porous frameworks and enhances energy storage performance.^[^ ^110^ ^]^ The reason for electrolyte‐wettability improvement is that semi‐ionic C—F bonds enable the surrounding area of F atoms more electronegative, which changing adsorption energy to tetraethylammonium cation (TEA^+^) from −48.97 kJ mol^−1^ for pure carbon surface model to −67.52 kJ mol^−1^ for fluorinated carbon surface mode (Figure 6b). The FNC‐4 electrode materials deliver a high specific capacitance of 168 F g^−1^ at a current density of 0.5 A g^−1^, and retains a high value of 153 F g^−1^ when the current density increases to 10 A g^−1^ (Figure 6c). It is found that FNCs with more F contents exhibit smaller ohmic resistance and *R*_ct_, which might be due to the improved electrolyte‐wettability on carbon materials surface (Figure 6c). Moreover, the influence of other atomic doping elements, such as N, S, P, and so on, on the electrolyte‐wettability and energy storage performance of carbon‐based electrode materials in organic electrolyte needs further investigation, because other atomic doping increasing surface energy and changing charge distribution and spin density except yielding weak polarity on the surface of carbon material may also improve the electrolyte‐pilicity and energy storage performance in organic electrolyte.^[^ ^47^ ^]^

##### Toward Ionic Liquid Electrolyte

**Figure 7:**
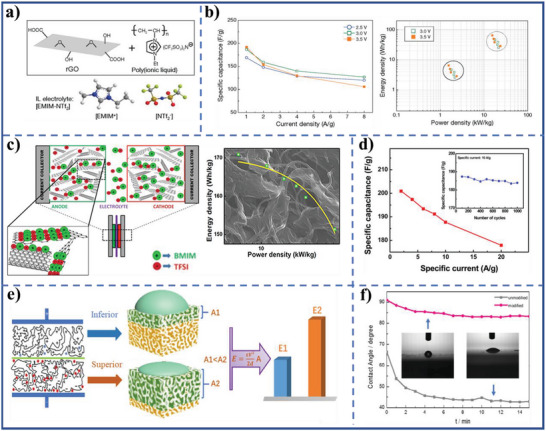
a) Schematic diagram of rGO, molecular formula of poly(ionic liquid), and cationic and anionic model of ionic liquid electrolyte. b) Plot of specific capacitance versus the current density and energy density versus power density at operating voltages of 3.0 and 3.5 V (the specific capacitance value is obtained from the galvanostatic charging–discharging (GCD) measurement. The energy and power density are normalized to the total mass of the two electrodes employed including the electrolyte and current collector (solid circle) and the mass of two poly(ionic liquid) modified rGO materials (dashed circle)). Reproduced with permission.^[^ ^111^ ^]^ Copyright 2011, American Chemical Society. c) Schematic illustration and of the functionalized multiwalled carbon nanotube/hydrogen exfoliated graphene/1‐butyl‐3‐methylimidazolium bis(trifluoromethyl sulfonyl)imide (f‐MWNTs/HEG/[BMIM] [TFSI]) ternary nanocomposite electrode and Ragone plot of f‐MWNTs/HEG/[BMIM] [TFSI] nanocomposite‐based supercapacitors. d) Specific capacitance of the f‐MWNTs/HEG/[BMIM] [TFSI] nanocomposite as a function of specific current (inset: cyclic stability of the nanocomposite at specific current of 10 A g^−1^). Reproduced with permission.^[^ ^112^ ^]^ Copyright 2012, American Chemical Society. e) Schematic diagram of the dominant role of electrolyte‐wettability in the utilization of electrode surface area (inferior electrolyte‐wettability leads to a small effective specific surface area (A1), resulting in a low energy density (E1). The enhancement of electrolyte‐wettability gives rise to an increase of the utilization of surface area (A2) and the energy density of supercapacitors (E2)), and f) the CA of the 1‐ethyl‐3‐methylimidazolium tetrafluoroborate ionic liquid droplet on the sample unmodified and modified by paraffin. Reproduced with permission.^[^ ^59^ ^]^ Copyright 2019, KeAi Publishing Communications Ltd.

Ionic liquid is known to exhibit moderately high ion conductivity, nonvolatility, high decomposition temperatures, and wide electrochemical stability windows, and many ionic liquids are being considered as electrolytes to increase supercapacitor operating voltages.^[^ ^111^ ^]^ However, electrolyte‐wettability is a big challenge in ionic liquid electrolyte‐based supercapacitors with carbon‐based materials as electrodes active materials, which is attributed to high viscosity and surface energy of the ionic liquid. The N, O codoping approach could improve electrolyte‐wettability of carbon‐based materials toward hydrophilic ionic liquid electrolyte, such as 1‐ethyl‐3‐methylimidazolium tetrafluoroborate (EMIMBF_4_).^[^ ^113^ ^]^ The N, O codoped carbon‐based material as supercapacitors electrodes materials exhibits an outstanding specific capacitance of 201 F g^−1^ in EMIMBF_4_ electrolyte at 0.5 A g^−1^ with a maximum energy density of 111 Wh kg^−1^. Based on the principle of similar polarity compatibility, the poly(ionic liquid)‐modified rGO by noncovalent grafting (**Figure** **7**a) could offer enhanced electrolyte‐wettability with certain ionic liquid electrolytes and promoted accessibility of ionic liquid electrolyte ions into the modified rGO electrode materials.^[^ ^111^, ^114^ ^]^ The poly(ionic liquid) modified rGO as an electrode material in supercapacitors incorporating ionic liquid electrolytes such as 1‐ethyl‐3‐methylimidazolium bis(trifluoromethylsulfonyl)amide (EMIM‐NTf_2_) exhibits a specific capacitance of 187 F g^−1^, an energy density of 6.5 Wh kg ^−1^, and a power density of 2.4 kW kg^−1^ (Figure 7b).^[^ ^111^ ^]^ To overcome that the cations of ionic liquid are immobilized by polymerization, grafting ionic liquid monomers by noncovalent, instead of poly(ionic liquid), can make both anion and cation mobile inside the electrode material under maintaining excellent electrolyte‐wettability of the carbon‐based electrode active materials (Figure 7c).^[^ ^112^ ^]^ This could enhance ion transport into the electrode materials by effectively increasing the number of ion diffusion paths. The specific capacitance of the ionic liquid monomers modified carbon‐based electrode materials at 2 A g^−1^ specific current are higher (201 F g^−1^) than the unmodified materials (Figure 7d). The maximum stored energy and maximum available power are extracted from Regone plot and found to be 170.66 Wh kg^−1^ and 148.43 kW kg^−1^, respectively (Figure 7c). The low time constant (0.575 s) and ESR (2.8 Ω) ensure the fast charging and discharging ability of the ionic liquid monomers modified carbon‐based electrode materials.

Because of relatively large viscosity and surface energy of ionic liquid in comparison to conventional electrolytes, some efforts are focused on reducing viscosity of the ionic liquid electrolyte by adding additive to improve electrolyte‐wettability of electrode materials in ionic liquid electrolyte (Figure 7e,f).^[^ ^59^, ^60^, ^115^, ^116^, ^117^, ^118^ ^]^ For example, an anionic surfactant ionic liquid (1‐butyl‐1‐methylpyrrolidinium docusate) being added in a nonsurfactant base ionic liquid (1‐propyl‐1‐methylpyrrolidinium bis(trifluoromethylsulfonyl) imide) as mixed ionic liquid electrolyte is used to improve electrolyte‐wettability of activated carbon electrode materials, which exhibits higher specific capacitance of 202 F g^−1^ at 150 °C and 10 mV s^−1^, compared to the nonsurfactant base ionic liquid of 160 F g^−1^.^[^ ^60^ ^]^

#### 3.1.2 Metallic Compound Electrode Active Materials for Supercapacitors

**Figure 8:**
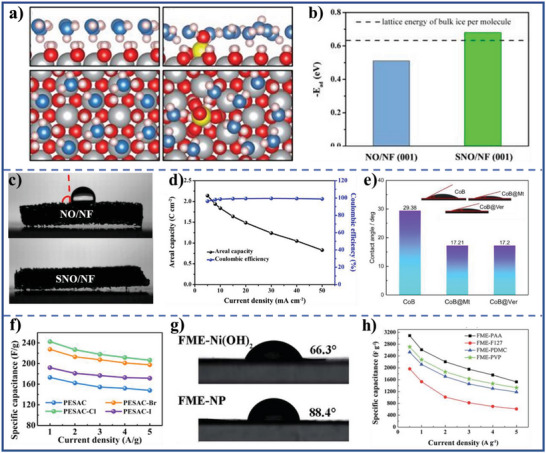
a) Water adsorption in hexagonal arrays and the most stable configuration of Ni(OH)_2_ on nickel foam (NO/NF) (001) (left) and sulfate functionalized Ni(OH)_2_ on nickel foam (SNO/NF) (001) (right) surfaces (gray, red, blue, pink, and yellow colors were used to represent Ni and O atoms in nickel hydroxides, O atoms in water, H atoms and S atoms, respectively). b) The calculated adsorption energies of water, c) water CA of NO/NF and SNO/NF, and d) areal capacity and coulombic efficiency of SNO/NF at serial current densities. Reproduced with permission.^[^ ^50^ ^]^ Copyright 2019, Wiley‐VCH. e) Electrolyte CA of CoB, CoB@Mt, and CoB@Ver. Reproduced with permission.^[^ ^71^ ^]^ Copyright 2022, Elsevier. f) Specific capacitances of PESAC (active carbon and PES acting as an electrochemically active material and a substrate material, respectively), PESAC‐X (X (X = Cl/Br/I)‐incorporated flexible electrode membrane) at different scan rates. Reproduced with permission.^[^ ^70^ ^]^ Copyright 2021, Elsevier. g) Water CA of the FME‐Ni(OH)_2_), and flexible membrane electrode no polymer (FME‐NP). Reproduced with permission.^[^ ^119^ ^]^ Copyright 2017, The Royal Society of Chemistry. h) Specific capacitance of the fabricated flexible membrane electrodes at different current densities (PAA‐*b*‐PAN‐*b*‐PAA modifying, F127 modifying, PDMC‐*b*‐PAN‐*b*‐PDMC modifying, and PVP‐*b*‐PAN‐*b*‐PVP modifying flexible membrane electrodes express as FME‐PAA, FME‐F127, FME‐PMDC, and FME‐PVP, respectively). Reproduced with permission.^[^ ^120^ ^]^ Copyright 2017, The Royal Society of Chemistry.

Compared with carbon‐based electrode active materials, metallic compound electrode active materials have higher specific capacity and energy density, owe to the energy storage mechanism (generally referring to pseudocapacitive and/or faradaic reaction) of metallic compound electrode active materials.^[^ ^121^, ^122^ ^]^ However, the inferior electrolyte‐wettability of metallic compound electrode materials severely confines exposure of the active site and electrolyte ionic diffusion, and further constraints the amount and kinetics of faradaic redox reactions on the interior surface of the electrode materials. By experimental study and density functional theory calculation, the incorporating a small amount of sulfate substantially raises the adsorption energy of water on *β*‐Ni(OH)_2_ (001) surface to above the lattice energy of bulk ice (0.68 eV > 0.63 eV) (**Figure** **8**a,b), rendering the high electrolyte‐wettability of the electrode material surface (with water CA approaching 0°) (Figure 8c).^[^ ^50^ ^]^ The areal capacities, rate capability, and coulombic efficiency of the resulting electrodes materials, and power density and energy density of the hybrid device are evaluated by recording its GCD curves at serial current densities from 5 to 50 mA cm^−2^ (Figure 8d). The sulfate functionalized Ni(OH)_2_ shows 9.3 times higher areal capacity and 1.8 times higher rate capability than the sulfate‐free control sample, and retains 81.3% capacity after 5000 cycles. When the sulfate functionalized Ni(OH)_2_ is used as the cathode active materials in a hybrid supercapacitors, the sulfate functionalized Ni(OH)_2_||activated carbon system achieves >95% coulombic efficiency, maximum energy density of 3.59 Wh m^−2^, and maximum power density of 44.63 W m^−2^, outperforming most of the existing Ni‐based supercapacitors. The extraordinarily hydrophilic clay materials such as vermiculite (Ver) and montmorillonite (Mt) are coated on CoB surface through the one‐step chemical liquid‐phase reduction method. The electrolyte‐wettability of CoB@Ver and CoB@Mt electrode materials exhibits significant improvement, resulting in a short relaxation time with a satisfactory specific capacitance (Figure 8e).^[^ ^71^ ^]^

For a flexible electrode, VN acting as an electrode active material, PES acting as a substrate material, the inferior electrolyte‐wettability toward aqueous electrolyte caused from hydrophobic PES could be ameliorated by a facile method based on introducing hydrated halide clusters into PES substrate. The modified flexible electrode exhibits the enhancement of energy storage performance (Figure 8f).^[^ ^70^ ^]^ Moreover, the flexible electrode membrane (nano‐nickel hydroxide acting as an electrochemically active material) with a membrane thickness of 60 mm is modified by grafting amphiphilic block copolymer onto the PES substrate surface by noncovalent bonding, which also demonstrates superior electrolyte‐philcity and the highest specific capacitance of 3, 090 F g^−1^ at a current density of 0.5 A g^−1^ (Figure 8g,h).^[^ ^119^, ^120^ ^]^ However, there is a note fact that the study on the electrolyte‐wettability of metallic compound electrode active materials is very rare. This may be due to the inherent polarity of metallic compound originating from the electronegative difference between metal and nonmetal.^[^ ^123^, ^124^, ^125^ ^]^ In addition, the high surface energy of amorphous region of metallic compound and dangling bonds for the edge region could also contribute to electrolyte‐wettability of metallic compound electrode.

#### 3.1.3 Conducting Polymer Electrode Materials for Supercapacitors

**Figure 9:**
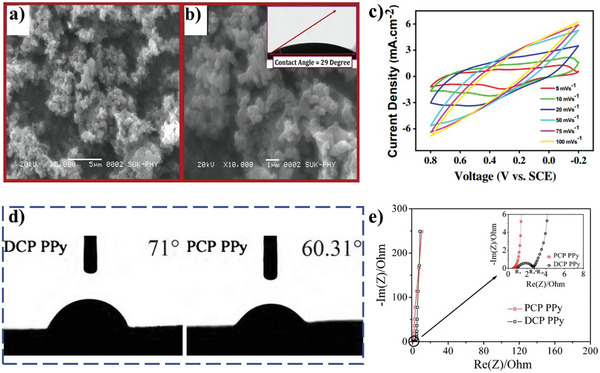
SEM images of PANI thin film at a) ×5000 and b) ×10 000 magnifications (inset shows water CA of PANI thin film), and c) CV curves of PANI thin film electrode at various scan rates in 1 m H_2_SO_4_ electrolyte. Reproduced with permission.^[^ ^45^ ^]^ Copyright 2013, Elsevier. d) The CA of water on DCP PPy and PCP PPy films and e) electrochemical impedance spectroscopy of PCP PPy and DCP PPy films in 3 m KCl aqueous solution (PCP PPy films: *R*_s_ = 0.52 Ω, *R*_ct_ < 0.1 Ω; DCP PPy films: *R*_s_ = 1 Ω, *R*_ct_ = 1 Ω). Reproduced with permission.^[^ ^126^ ^]^ Copyright 2010, Elsevier.

Conducting polymer as electrode active materials for supercapacitors has many advantageous properties, including high theoretical capacitance (2000 F g^−1^ of PANI), ease of synthesis, low cost, good electronic conductivity, and environmental benignity. However, the unfavorable electrolyte‐wettability toward aqueous electrolytes confines its practical capacitance.^[^ ^127^ ^]^ The prepared nanostructure PANI by chemical oxidization followed microwave assistance^[^ ^45^, ^128^ ^]^ and potentiostatic deposition^[^ ^129^, ^130^, ^131^ ^]^ exhibits admiring electrolyte‐wettability within aqueous electrolyte, being attributed to high surface energy of nanocrystalline and amorphous, and the strong cohesive force between polar functional groups and hydroxide presented in the PANI and aqueous electrolyte. For example, Lokhande and co‐workers report that the PANI thin film shows low water CA of 29° (**Figure** **9**a,b) and high specific capacitance of 546 F g^−1^ at the scan rate of 5 mV s^−1^ (Figure 9c).^[^ ^45^ ^]^ By potentiostatic deposition method, the polypyrrole (PPy) thin film with excellent electrolyte‐wettability is also prepared as electrode active materials for supercapacitors, holding high specific capacitance of 381 F g^−1^ in 0.5 m Na_2_SO_4_ aqueous solution at scan rate 5 mV s^−1^.^[^ ^132^ ^]^ Compared with direct current polymerization (DCP), the synthesized PPy film by pulse current polymerization (PCP) could generate shorter molecular chains and less —CH_2_— and —CH_3_ groups, leading to improving electrolyte‐wettability by aqueous electrolyte and promoting diffusion of electrolyte ions within the PPy film (Figure 9d).^[^ ^126^ ^]^ The resulting materials demonstrates high specific capacitance and charging and discharging rate (the specific capacitance reaches 260 F g^−1^ at charging and discharging current of 20 A g^−1^ in 3 m KCl aqueous solution) and very small *R*_ct_ (*R*_ct_ < 0.1 Ω) (Figure 9e). PANI being dispersed on the surface of hydrophilic substrates (such as polyvinyl alcohol) enables better wetting of aqueous electrolyte and more effective ionic transport inside the electrode materials to enhance energy storage performance of PANI.^[^ ^133^ ^]^ Furthermore, the electrolyte‐wettability of the composite of conducting polymer with carbon materials is improved by generally enhancing the electrolyte‐wettability of the carbon materials.^[^ ^127^ ^]^

Excellent electrolyte‐wettability of electrode could increase ion‐accessible surface area of the electrode, facilitate ion diffusion in the electrode channels, and promote ion movement on the electrode surface. Thus, the impact of improving electrolyte‐wettability of electrode on the energy storage performance of the electrode for surpercapacitors would generally be summarized in four aspects: i) increase specific capacitance of the electrode, ii) enhance rate performance of the electrode, iii) reduce the impedance, especially *R*_ct_ of the electrodes, and iv) augment energy density and power density of the supercapacitors that are assembled by the electrode. However, some negative effects of improving electrolyte‐wettability of electrode should be fully considered in future, for example, the introduction of polar functional groups, molecular brushes, and polymer brushes may lead to a decrease in electrode conductivity, and grafting polymer brushes may increase steric resistance of electrolyte ion transport within electrode.

### 3.2 Electrolyte‐Wettability in Metal Ion Batteries

**Figure 10:**
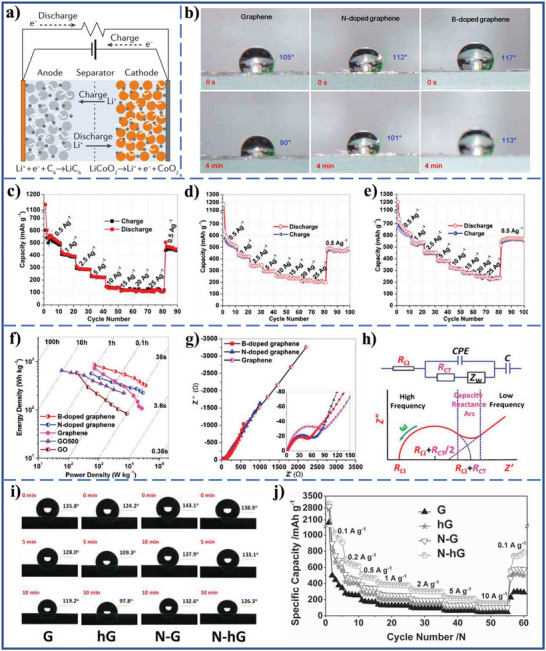
a) The basic configuration and working mechanism of a battery (a typical battery device consists of an anode and cathode film sandwiched between two current collectors and isolated by an insulating separator). Reproduced with permission.^[^ ^134^ ^]^ Copyright 2019, Springer Nature. b) Photographs of water droplets resting on the pristine graphene, the N‐doped graphene, and the B‐doped graphene films for 0 s and 4 min, showing that the pristine graphene sheets have better wettability toward water than doped graphene sheets. Rate capabilities and cycle performance of c) the pristine graphene, d) N‐doped graphene, and e) B‐doped graphene electrodes obtained over a wide range of high current densities, from 0.5 to 25 A g^−1^. f) Ragone plots for the pristine graphene, N‐doped graphene, B‐doped graphene, graphene oxide (GO), and GO500 (GO500 is prepared by thermal reduction of GO at 500 °C for 2 h) based cells with lithium metal as the counter/reference electrode (the calculation of gravimetric energy and power density is based on the active material mass of a single electrode). g) Nyquist plots of pristine, N‐doped, and B‐doped graphene (inset, high‐frequency region Nyquist plots). h) Modeled equivalent circuit and schematic of EIS, where *R*_Ω_ stands for the electrolyte resistance, *R*_CT_ the charge transfer resistance, ZW the “Warburg”‐type element related to Li^+^ diffusion, CPE the constant phase element, and the potential‐dependent capacitance. Reproduced with permission.^[^ ^27^ ^]^ Copyright 2011, American Chemical Society. i) A typical photograph of water droplets resting on G, hG, N‐G, and N‐hG electrodes for 0, 5, and 10 min (the CA measurements are carried out by three times and the average values of CA are presented). j) Rate capability of G, hG, N‐G, and N‐hG measured in the voltage range of 0.02–2.5 V. Reproduced with permission.^[^ ^28^ ^]^ Copyright 2015, Wiley‐VCH.

In the past few decades, Li^+^ batteries and other metal ion batteries, such as sodium ion (Na^+^), potassium ion (K^+^), and magnesium ion (Mg^2+^) batteries have been widely studied and successfully used as the rechargeable energy source of choice in various portable and smart devices and electric vehicles because of their relatively high energy density and power density, as well as long lifetime.^[^ ^135^, ^136^ ^]^ In a metal ion battery, the metal ions are transported and ejected/inserted in electrode where redox reactions occur within the active materials of electrode at a given electrochemical potential. Therefore, the energy is stored in the bulk volume of the electrode, which enables high energy densities of the electrode (≥100 Wh kg^−1^) (**Figure** **10**a).^[^ ^134^ ^]^ Nevertheless, the electrodes used in metal ion batteries have usually inefficient rate capability, and always suffer from low capacities at rapid charging and discharging rates.^[^ ^137^ ^]^ The reason is that the electrolyte‐wettability of electrode brings about a significant effect on the utilization rate of electrode active material, the transport of electrolyte ion in the electrode channels, and the distribution of charge on the surface of the electrode, which affects energy storage performance of the electrode for metal ion batteries. Thus, constructing the electrode with excellent electrolyte‐wettability could effectively increase specific capacitance and rate capability, and decrease impedance of the electrode for metal ion batteries based on increasing the utilization rate of electrode active materials, enhancing the fast diffusion of electrolyte ions, and facilitating the movement of electrolyte ions on the electrode surface. In view of the above, the effect of electrolyte‐wettability on electrochemical energy storage performance of the electrodes that are applied in alkali metal ion batteries (including Li^+^, Na^+^, and K^+^) and Mg^2+^ batteries is reviewed in this section. In metal ion batteries, the electrolyte‐wettability of electrode is improved mainly by modifying electrode active materials and coating layer.

#### 3.2.1 Carbon‐Based Electrode Materials for Anode of Metal Ion Batteries

Carbon‐based electrode active materials are widely applied as anodes of alkali metal ion batteries for energy storage devices due to their high surface area, excellent electrical conductivity, and extraordinary electrochemical inertness.^[^ ^138^, ^139^, ^140^, ^141^, ^142^ ^]^ The introduction of polar atoms (for example, B, N, S, and P) is identified as the most promising method to enhance electrolyte‐wettability of the electrode active materials, which can accelerate the fast diffusion of electrolyte ion within the electrode and electrode/electrolyte interface reaction.^[^ ^141^ ^]^

Some N‐ or B‐doped graphenes have worse wettability toward water than the pristine graphene, while the N‐ or B‐doped graphenes should have better electrolyte‐wettability toward organic electrolyte used in Li^+^ batteries than the pristine graphene. This reason is that after some O‐containing functional groups with strong polarity are removed during the N‐ or B‐doping (8.55% O content for the pristine graphene, 3.13% O content for the N‐doped graphene, and 6.06% for the B‐doped graphene), the N‐ or B‐doped graphenes with weak polarity is more compatible with nonpolar organic electrolytes (Figure 10b).^[^ ^27^ ^]^ The improved electrolyte‐wettability toward organic electrolyte would promote electrolyte ion movement in the electrode/electrolyte interface and facilitate electrolyte ion diffusion in the interior of the electrode, thus the N‐ and B‐doped graphenes as anode active materials for Li^+^ batteries show superior charging and discharging performance. As shown in Figure 10c–e, the samples are first charged and discharged at 0.5 A g^−1^ for 10 cycles and then the current rate increases stepwise to as high as 25 A g^−1^, for 10 cycles at each rate. The N‐ and B‐doped graphene exhibits very high capacity and good stability at high current rates, different from the capacity fluctuations of the pristine graphene. Examples include higher reversible capacities of 1043 mAh g^−1^ for the N‐doped graphene (Figure 10d) and 1549 mAh g^−1^ for the B‐doped graphene (Figure 10e) than 955 mAh g^−1^ for the pristine graphene (Figure 10c) at a low current rate of 50 mA g^−1^, very fast charging rate of ≈28 s (126 C) for the N‐doped graphene and ≈33 s (106 C) for the B‐doped graphene, higher power densities, and energy densities of the N‐doped graphene (≈29.1 kW kg_electrode_^−1^ and ≈226 Wh kg_electrode_^−1^) and the B‐doped graphene (≈34.9 kW kg_electrode_^−1^ and ≈320 Wh kg_electrode_^−1^) than those of the pristine graphene (26.3 kW kg_electrode_^−1^ and 106 Wh kg_electrode_^−1^) at 25 A g^−1^ (Figure 10f), and much lower electrolyte resistances (*R*_Ω_ = 1.87 and 1.32 Ω for the N‐ and B‐doped graphene, respectively) and *R*_ct_ (*R*_ct_ = 63.99 and 59.98 Ω for the N‐ and B‐doped graphene, respectively) than those of the pristine graphene (*R*_Ω_ = 2.25 Ω, *R*_ct_ = 87.33 Ω) (Figure 10g,h). The graphene with a large amount of holes in its planar sheet (hG) is prepared by directly heating commercially available graphene in air to deliver a high energy (gravimetric/volumetric) density and high rate capability for Li^+^ batteries.^[^ ^28^, ^143^ ^]^ However, the lower N content and higher O‐containing functional groups of the hG originating from high temperature oxidation of air in the process of forming holes possess hydrophilicity, which decreases the electrolyte‐wettability toward organic electrolyte (Figure 10i).^[^ ^28^ ^]^ N‐doped graphene (N‐G) and N‐doped holey graphene (N‐hG) are prepared by annealing graphene (G) and hG at 500 °C for 5 h in a tube furnace, under a mixed NH_3_/Ar gas (flow rate of NH_3_:Ar = 3:7300 sccm), respectively. According to the results of the water CA test (Figure 10i), N‐doping enhances the hydrophobicity of hG and G, and hence enhances the electrolyte‐wettability toward organic electrolyte for N‐G and N‐hG, because of the partial removal of O‐containing functional groups through the doping‐induced reduction process. The improved electrolyte‐wettability of the N‐hG also enhances the fast diffusion of Li^+^ with a low ion‐transport resistance and maximizes the effective storage of Li^+^ during the discharge–charge processes. Thus, the N‐hG anode active materials for Li^+^ batteries exhibit the highest initial charging and discharging capacity of 989.5 mAh g^−1^ at 0.1 A g^−1^, followed by the N‐G (852.3 mAh g^−1^), and then the hG (590.4 mAh g^−1^) and G (368.2 mAh g^−1^). Moreover, a higher reversible capacity and rate capability of the N‐hG anode active materials are also verified by increasing the current density from 0.2 to 10 A g^−1^ and then back to 0.1 A g^−1^ (Figure 10j). Note that decrease of O content in carbon anode for Li^+^ batteries could improve its electrolyte‐wettability toward organic electrolyte, but the effect of doping other heteroatom on electrolyte‐wettability toward organic electrolyte still needs further exploration. The electrolyte‐wettability of graphite anode materials could be improved by surface coating amorphous Al_2_O_3_. Wettability test and full battery tests adopting LiCoO_2_ cathode and Al_2_O_3_‐coated graphite anodes reveal that the fast‐charging improvement results from the increased electrolyte‐wettability of the Al_2_O_3_‐coated graphite that is induced by the amorphous Al_2_O_3_ layer coating on its surface.^[^ ^144^ ^]^ Besides, the C@MnO core–shell composites and the carbon‐coated Bi_2_O_3_ nanoparticles distributed on N‐doped reduced graphene oxide (Bi_2_O_3_@C@N‐G) composites are applied in Li^+^ batteries, the porous carbon shell deriving from phenolic resin and N–G are beneficial to improving electrolyte‐wettability of the entire C@MnO and Bi_2_O_3_@C@N‐G composites, respectively, which enhance energy storage performance of the composites.^[^ ^145^, ^146^ ^]^

**Figure 11:**
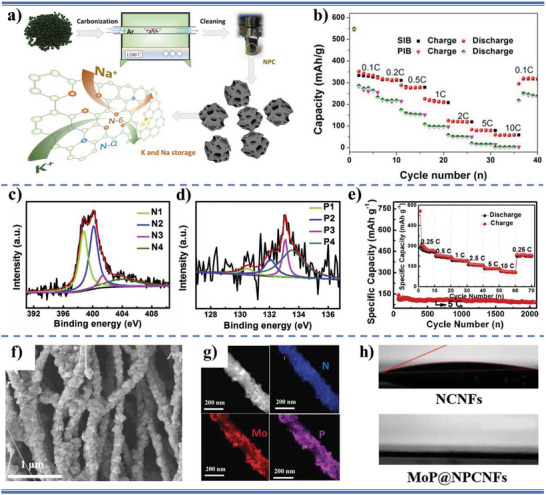
a) Schematic diagram of the synthesis process and ions transport of N‐doped porous carbon anode materials; and b) rate capacity performance of N‐doped porous carbon in K^+^ batteries and Na^+^ batteries at different current rates. Reproduced with permission.^[^ ^142^ ^]^ Copyright 2019, Elsevier. High‐resolution XPS spectra of c) N1s and d) P2p of N, P codoped carbon sheets. e) Long‐term cycling performance of N, P codoped carbon sheets anode over 2000 cycles at a rate of 5 C after 70 cycles rate performance measurement (the inset is the rate performance of N, P codoped carbon sheets at a variety of current rates). Reproduced with permission.^[^ ^29^ ^]^ Copyright 2018, Elsevier. Morphological characterizations of the MoP@NPCNFs nanocomposite membrane: f) SEM image and g) scanning transmission electron microscope (STEM) image and the corresponding N, Mo, and P elemental mapping images of MoP@NPCNFs and h) the CA testing results of NCNFs and MoP@NPCNFs. Reproduced with permission.^[^ ^72^ ^]^ Copyright 2015, Wiley‐VCH.

Li^+^ batteries in large‐scale practical applications may be hampered by the limited Li resource reserves (20 ppm in the earth cluster).^[^ ^147^ ^]^ In consideration of this, Na^+^ batteries and K^+^ batteries are promising potential substitutes for Li^+^ batteries owing to similar physiochemical properties in the same main group and the rich resource of Na (23 600 ppm) and K (20 900 ppm).^[^ ^48^, ^141^ ^]^ For carbon anode materials used for Na^+^ and K^+^ batteries, the incorporation of polar atoms (e.g., B, N, S, and P) is considered to be the most promising method to enhance the electrolyte‐wettability of the carbon electrode material surface. In addition to forming a similar polarity to organic electrolytes by removing O‐containing functional groups, the mechanisms by which polar atom doping enhances electrolyte‐wettability toward organic electrolytes generally include three aspects: i) polar atom doping increase surface defect of the carbon materials, resulting in high surface energy of the doped carbon materials;^[^ ^47^ ^]^ ii) polar atom doping gives rise to high binding energy with electrolyte ions from organic electrolytes;^[^ ^96^ ^]^ and iii) the peculiar surface charge distribution of the polar atom doped carbon materials, enable cation‐philicity of the negatively charged atoms and anion‐philicity of the positively charged atoms in organic electrolyte.^[^ ^95^ ^]^ Thus, the N‐doped porous carbon synthesized by a direct pyrolysis of rich‐N precursors (**Figure** **11**a) could provide good electrolyte‐wettability and numerous ion transport. When N‐doped porous carbon is used as anode for Na^+^ and K^+^ batteries, it presents better rate performance (121 mAh g^−1^ for Na^+^ batteries and 51 mAh g^−1^ for K^+^ batteries at the current densities of 2 C) (Figure 11b).^[^ ^142^ ^]^ The N, P codoped carbon sheets (Figure 11c,d) holding preferable electrolyte‐wettability as anode for Na^+^ batteries would present a discharging capacity of 233, 204, 177, and 143 mAh g^−1^ at a rate of 0.5 C (0.1 A g^−1^), 1 C (0.2 A g^−1^), 2.5 C (0.5 A g^−1^), and 5 C (1 A g^−1^). Even at a high rate of 5 C, a stable specific capacity of 105 mAh g^−1^ still could be delivered after 2000 cycles, suggesting a superior rate capability and excellent cycle stability (Figure 11e).^[^ ^29^ ^]^ Constructing nanoalloy/carbon composites via 3D porous carbon as a substrate for K^+^ batteries exhibit high specific capacity while prevents the aggregation of the alloy and releases volume expansion.^[^ ^148^ ^]^ However, electrolyte‐wettability is a key factor that may affect the diffusion kinetics of K^+^ in the 3D porous substrate and the rate performance of K^+^ batteries. The N‐doped 3D carbon network confined nanosized SnSb alloy (3D SnSb@NC) coupling with an ether‐based electrolyte (0.5 m KPF_6_ in 1,2‐dimethoxyethane (DME) solvent) displays much better electrolyte‐wettability than the ethylene carbonate (EC)/dimethyl carbonate (DMC)‐based electrolyte (0.8 m KPF_6_ dissolved in mixture solutions of 1:1 (weight ratio) EC–DMC).^[^ ^48^ ^]^ The 3D SnSb@NC electrode coupled with an optimized DME‐based electrolyte for K^+^ batteries deliver a high reversible capacity of 185.8 mAh g^−1^ after 200 cycles at 500 mA g^−1^ and the corresponding capacity in the EC/DMC‐based electrolyte is only 147.4 mAh g^−1^, indicating a high‐rate cycling performance of the 3D SnSb@NC electrode coupled with the DME‐based electrolyte. The 3D N‐doped carbon nanofibers (NCNFs) are synthesized by carbonizing ZIF‐8/PAN polymer nanofiber cloth.^[^ ^72^ ^]^ After adding Mo phases into NCNFs by immersing the NCNFs membrane into a saturated ammonium molybdate solution, the N, P codoped carbon nanofibers decorated with MoP ultrafine nanoparticles (MoP@NPCNFs) (Figure 11f,g) are obtained via phosphorization treatment of Mo@NCNFs with NaH_2_PO_2_ at 600 °C in the H_2_–Ar mixture atmosphere. MoP@NPCNFs shows better electrolyte‐wettability than NCNFs (Figure 11h), because the MoP ultrafine nanoparticles confer high surface energy on the surface of MoP@NPCNFs. Thus, the MoP@NPCNF flexible membrane as anode for K^+^ batteries demonstrates outstanding K‐storage performance with a high capacity of 320 mAh g^−1^ at 100 mA g^−1^, a superior rate capability maintaining 220 mAh g^−1^ at 2 A g^−1^, as well as a capacity retention of more than 90% even after 200 cycles.

#### 3.2.2 Metallic Oxide for Cathode of Metal Ion Batteries

**Figure 12:**
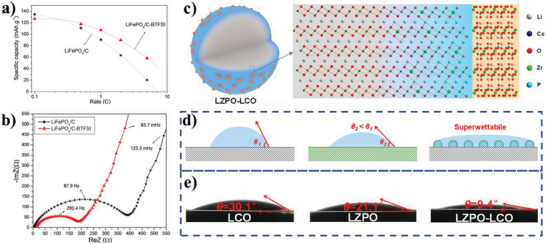
a) Rate capability and b) electrochemical impedance spectroscopy measurements for unmodified LFP/C and LFP/C‐BTFSI electrodes. Reproduced with permission.^[^ ^73^ ^]^ Copyright 2015, American Chemical Society. c) Schematic micromorphology and crystal structure of LZPO–LCO. d) Schematic illustration of how LZPO–LCO achieves superwettability. e) The contact angle of each sample with the electrolyte at room temperature. Reproduce with permission.^[^ ^74^ ^]^ Copyright 2023, American Chemical Society.

LiFePO_4_ (LFP) as a cathode material for Li^+^ batteries is extensively concerned in the last few decades. Carbon coating could not only improve electrical conductivity, mitigate volume expansion, and avoid direct contact of LFP with electrolytes, but also could enhance electrolyte‐wettability of LFP (because of the similar polarity of graphitic carbon with the electrolyte: 1 m LiPF_6_ in EC/DEC 2:1).^[^ ^149^ ^]^ The PEA‐coated LFP shows better electrolyte‐wettability than carbon‐coated LFP, because the PEA ether–O structure is chemically similar to EC and DEC. Potentiostatic measurements show that the capacity of the PEA‐coated LFP is 12% higher than that of carbon‐coated LFP in the organic electrolytes. A family of aryl‐trifluoromethanesulfonylimide (such as BTFSI) moiety is grafted on carbon‐coated LFP by covalent bond, which also promotes the electrolyte‐wettability of the modified‐electrode and enhances Li^+^ diffusion at the active electrode material/electrolyte interface, while not affects the electrical conductivity of the electrode.^[^ ^73^ ^]^ Two‐electrode electrochemical coin batteries are assembled with Li metal anodes, Celgard‐2320 separator, 1 m LiPF_6_ in EC/DEC/DMC (1:1:1 by vol) electrolyte, and LFP/C and/or LFP/C‐BTFSI cathode. As shown in **Figure** **12**a, the LFP/C‐BTFSI shows better rate performance (at 5 C, the LFP/C‐BTFSI retains 46.1% of its initial discharging capacity at 0.1 C) and lower *R*_ct_ (198 Ω) than the bare LFP/C (only 14.8%, 410 Ω) (Figure 12b). Besides, the current LiCoO_2_ (LCO) cathode still shows low practical capacity and suffers insufficient low‐temperature performance, which most likely originates from the poor electrolyte‐wettability. Huang and co‐workers enhance electrolyte‐wettability of LCO by coating a more electrolyte‐wettable materials and constructing a specific surface microstructure.^[^ ^74^ ^]^ As a precursor material, Li^+^ conductive Li_2_Zr(PO_4_)_2_ (LZPO) oligomer could stick to LCO uniformly, which partially reacts with the shallow surface LCO to form a Zr‐doped LCO layer at high temperature. Meanwhile, the other part of the LZPO oligomer forms LZPO nanoparticles interspersed on the LCO surface, exhibiting a massage‐ball‐like morphology (Figure 12c). Figure 12d illustrates the mechanism of how the LCO interspersed massage‐ball‐like LZPO (LZPO–LCO) achieves superwettability. The electrolyte‐wettability will be enhanced significantly under double action of chemical composition (the more wettable LZPO than LCO) and geometric structure (massage‐ball‐like LZPO shows high roughness). The CAs of LCO, LZPO, and LZPO–LCO with organic electrolyte are 30.1°, 21.1°, and 9.4° (Figure 12e), respectively, which verify the practical rationality of the mechanism. The LZPO–LCO electrode shows an ultrahigh capacity of ≈200 mAh g^−1^ at 0.2 C, and the fabricated full cells deliver a high energy density of ≈340 Wh kg^−1^ at −25 °C.

### 3.3 Electrolyte‐Wettability in Metal‐Based Batteries

The limiting theoretical capacity of metal ion batteries cannot fulfill the growing demand of large electric facilities like electric vehicle, which motivates the revival of metal‐based batteries due to their high theoretical specific capacity (3860 mAh g^−1^ for Li metal anode, 1165 mAh g^−1^ for Na metal anode, 687 mAh g^−1^ for K metal anode, 820 mAh g^−1^ for Zn metal anode).^[^ ^77^, ^150^, ^151^, ^152^ ^]^ However, the formation and growth of dendrites on metal anodes during continuous metal deposition and striping can severely penetrate through the separator and induce short‐circuiting in a rechargeable batteries system, which lead to the safety issue in metal‐based rechargeable batteries.^[^ ^96^ ^]^ Developing electrolyte‐wettable metal anode and current collector (for the anode‐free metal batteries consisting of a cathode and a bare current collect at anode side, in the process of discharging, the current collectors undergo the same process of interfacial metal deposition as metal anode^[^ ^153^ ^]^) for dispersing local current density and regulating active metal nucleation sizes and sites during electrodeposition could be a promising strategy to resolve the issue mentioned above. Furthermore, the carbonaceous matrix of cathode with well electrolyte‐wettability could also ensure fast ion transport in the cathode for alkali metal batteries (such as alkali metal–O_2_ batteries, alkali metal–CO_2_ batteries, lithium–sulfur (Li–S) batteries, and so on). In view of the above, in this section, the effect of electrolyte‐wettability on electrochemical energy storage performance of the electrodes applied in alkali metal batteries (including Li, Na, and K metal) and Zn–metal batteries are reviewed.

#### 3.3.1 Carbon‐Based Electrodes for Alkali Metal Batteries

##### Carbon‐Based Anode Current Collectors

**Figure 13:**
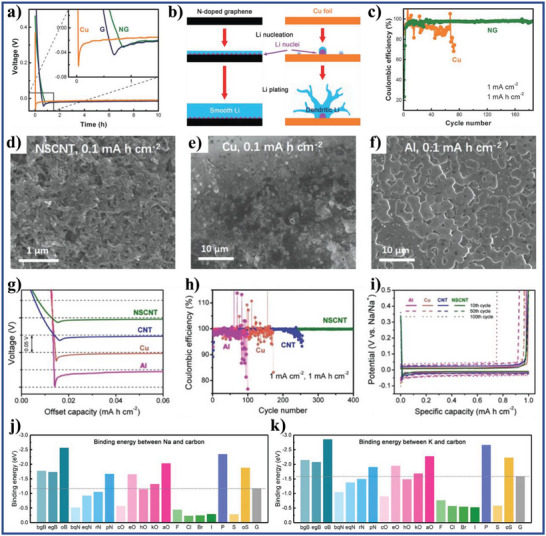
a) The voltage–time curves during Li nucleation at 0.05 mA cm^−2^ on Cu foil, graphene (G), and N‐doped graphene (NG) electrodes. b) Schematic representation of the Li nucleation and plating process on NG electrode and Cu foil electrode and c) coulombic efficiency of Cu foil and NG electrode with a cycling capacity of 1.0 mAh cm^−2^, at current density of 1.0 mA cm^−2^. Reproduce with permission.^[^ ^57^ ^]^ Copyright 2017, Wiley‐VCH. d) Ex situ SEM images of Na deposits on d) NSCNT paper, e) Cu foil, and f) Al foil at a current density of 0.1 mA cm^−2^ with a nucleation capacity of 0.1 mA h cm^−2^. g) The voltage–capacity profiles during Na nucleation on different current collectors at a current density of 0.05 mA cm^−2^. h) Coulombic efficiencies of Na plating/stripping on Cu foil, Al foil, CNT paper, and NSCNT paper at a current density of 1 mA cm^−2^ with a capacity limitation of 1 mA h cm^−2^ and i) the corresponding voltage profiles of Na plating/stripping on different current collectors at 10th, 50th, and 100th cycles. Reproduce with permission.^[^ ^96^ ^]^ Copyright 2018, Wiley‐VCH. The summary of binding energy on carbon materials: j) the binding energy between Na and carbon and k) the binding energy between K and carbon. Reproduce with permission.^[^ ^154^ ^]^ Copyright 2020, Elsevier.

The electrolyte‐wettability of carbon‐based alkali metal anode current collectors is improved by changing the surface chemistry of the carbon‐based current collectors to enhance the mutual‐philicity with alkali metal cations (such as reducing the binding energy and adsorption energy between the two).^[^ ^154^ ^]^ A N‐doped graphene being Li^+^‐philicity is obtained by treating the graphene at 600 °C with NH_3_ flowing (200 mL min^−1^) for 4.0 h in a quartz tube and adopted as the Li deposition anode current collector to regulate Li metal nucleation and suppress dendrite growth.^[^ ^57^ ^]^ As a result, the N‐doped graphene anode current collect exhibits a low nucleation overpotential of only 0.022 V at the beginning of the Li deposition process (**Figure** **13**a), a dendrite free morphology during repeated Li deposition (Figure 13b), and a high coulombic efficiency of 98% at 1.0 mA cm^−2^ with a cycling capacity of 1.0 mAh cm^−2^ for near 200 cycles (Figure 13c). In addition, electrolyte‐wettable ZnO can offer much more uniform nucleation sites and smaller nucleation overpotential for Li deposition, thus the ZnO quantum dots decorated 3D hierarchical porous carbon current collector demonstrates dendrite‐free Li deposition and striping behavior.^[^ ^155^ ^]^

Na and K metal are expected to be a more abundant, cost‐effective, and sustainable alternative to Li as anode active materials in large‐scale electric vehicle and portable device applications for the future.^[^ ^154^, ^156^ ^]^ Inspired by the above studies on Li metal anodes, a N, S codoped carbon nanotube (NSCNT) paper is used as the current collector to control Na nucleation behavior and suppress the Na dendrite growth.^[^ ^96^ ^]^ The N‐ and S‐containing functional groups on the carbon nanotubes induce the NSCNT paper to be highly Na^+^‐philicity, which can guide the initial Na nucleation, reduce the local current density, and direct Na to distribute uniformly on the NSCNT paper (Figure 13d–f). The NSCNT paper current collectors as the working electrodes exhibits that the nucleation overpotential is only 9 mV, which is much smaller than those of Cu foil (27 mV) and Al foil (45 mV) (Figure 13g), the average coulombic efficiency of Na deposition and stripping at a current density of 1.0 mA cm^−2^ with a capacity limitation of 1.0 mAh cm^−2^ is ≈99.82% for 400 cycle (Figure 13h), the hysteresis of the voltage plateaus between Na deposition and stripping is only 20 mV (Figure 13i), and the lower resistance for metallic Na to nucleate on porous NSCNT paper. As a proof of concept, it is also demonstrated that the electrochemical performance of Na–O_2_ batteries using the Na/NSCNT anodes show significantly improved cycling performances compared with Na–O_2_ batteries with bare Na metal anodes. Zhang and co‐workers probe the Na^+^ and K^+^‐philicity of a series of B‐, N‐, O‐, F‐, P‐, S‐, Cl‐, Br‐, and I‐doped carbon‐based current collector by first‐principles calculations to afford a deep and insightful understanding of Na^+^‐philic and K^+^‐philic chemistry of Na and K anodes and establish general principles of designing highly Na^+^‐philic and K^+^‐philic carbon‐based current collector.^[^ ^154^ ^]^ The B‐doping includes graphitic boron in the bulk phase (bgB), graphitic boron on the edge (egB), and B‐2C‐O‐type boron (oB). The N‐doping includes quaternary nitrogen in the bulk phase (bqN), quaternary nitrogen on the edge (eqN), pyrrolic nitrogen (rN), and pyridinic nitrogen (pN). The O‐doping includes carboxylic group (aO), cyclic oxygen (cO), epoxy group (eO), hydroxyl group (hO), and ketone group (kO). The S‐doping includes sulfur (S) and sulfonyl group (oS). By summarizing the binding energies of Na/K on pristine and polar atom‐doped carbon materials (Figure 13j,k), the O‐doping,^[^ ^157^ ^]^ O, B codoping, O, S codoping, and O, P codoping strategy are predicted to be effective methods to improve the Na^+^ and K^+^‐philicity of carbon‐based current collector and render safe and dendrite‐free Na and K metal anodes.^[^ ^154^ ^]^ Furthermore, the local dipole of doping functional groups and charge transfer during Na/K deposition are regarded as key principles to reveal the Na^+^ and K^+^‐philicity nature of doping sites.

##### Carbon‐Based Cathode Substrates

**Figure 14:**
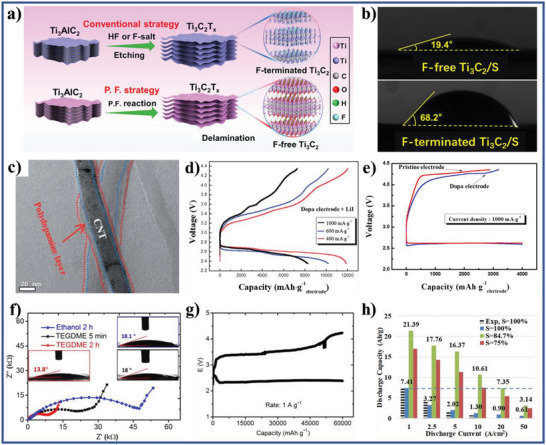
a) Schematic illustration for the fabrication of F‐terminated Ti_3_C_2_ via a conventional F‐containing strategy and P.F. strategy for the fabrication of F‐free Ti_3_C_2_. b) CA measurements between electrolyte and F‐free Ti_3_C_2_/S and F‐terminated Ti_3_C_2_/S electrodes. Reproduced with permission.^[^ ^77^ ^]^ Copyright 2022, American Chemical Society. c) Transmission electron microscope (TEM) image of polydopamine‐coated CNTs. d) Initial discharging–charging profiles of the dopa electrodes (the electrode employing polydopamine‐coated CNTs as the “dopa electrode” and the electrode employing uncoated CNTs as the “pristine electrode”) measured using LiI‐containing electrolyte at current densities of 400, 600, and 1000 mA g^−1^. Reproduced with permission.^[^ ^76^ ^]^ Copyright 2014, The Royal Society of Chemistry. e) Initial discharging–charging profiles of pristine and dopa electrodes measured using pristine electrolyte. Reproduced with permission.^[^ ^158^ ^]^ Copyright 2014, Springer. f) Comparison of CA and interface resistance of MWCNT with different treatments and g) full discharge/charge curves of Na–CO_2_ batteries with normal cathode at 1 A g^−1^ (the discharging and charging capacities equal to 60 000 mAh g^−1^ based on the mass of t‐MWCNTs). Reproduced with permission.^[^ ^159^ ^]^ Copyright 2016, Wiley‐VCH. h) Discharging capacities at different current rates when the electrode has different saturation levels. Reproduced with permission.^[^ ^160^ ^]^ Copyright 2018, American Chemical Society.

The Li–S batteries as a next generation high energy density storage system are to meet the demands of high storage energy and low cost.^[^ ^161^ ^]^ Encapsulating S/Li_2_S into carbon‐based substrates as cathode for Li–S batteries is an effective strategy to improve the electronic conductivity of the electrode and reduce the dissolution of polysulfide species. However, nonpolar carbon materials cannot efficiently suppress the dissolution and outward diffusion of polar Li polysulfide species via weak physical adsorption/confinement, resulting in unsatisfactory capacity utilization, rate capability, and long‐term cycling stability.^[^ ^162^ ^]^ Polar atom (such as O, N, S, and B) doping^[^ ^125^ ^]^ and polar materials coating^[^ ^52^, ^161^ ^]^ can enhance interaction between the carbon materials and the polysulfides, thus suppressing the notorious shuttle effect. In addition, these approaches may also ameliorate electrolyte‐wettability of carbon‐based S cathode, because of the higher electrolytic cation (Li^+^, Na^+^, and so on)‐philicity of the polar surface than nonpolar carbon surface, even though it is rarely reported in previous literature. Xiao and co‐workers fabricate a F‐free Ti_3_C_2_ with high purity of 95% through low temperature “soft chemistry” approach based on photo‐Fenton (P.F.) reaction and uses it as a substrate loaded with S for Li–S batteries (**Figure** **14**a).^[^ ^75^ ^]^ The F‐free Ti_3_C_2_/S cathode with a uniform S distribution and high S content of 69.6% exhibits substantially improves Li^+^ ion diffusion rate, which is mainly attributed to the fact that the absence of ‐F groups in F‐free Ti_3_C_2_ results in better electrolyte‐wettability (Figure 14b).

Due to high specific energy and the inexhaustible cathode O_2_ and CO_2_ from the ambient, the alkali metal–O_2_ and alkali metal–CO_2_ batteries are considered to be promising alternative to current rechargeable batteries.^[^ ^159^, ^160^ ^]^ In practice, the capacity of the carbon‐based O_2_ and CO_2_ cathode is proportional to the area of the two‐phase boundary between the electrolyte (containing alkali metal ions and dissolved O_2_ or CO_2_) and the electrode.^[^ ^162^ ^]^ Thus, to achieve the ideal electrode with fast electrolyte cation transport and high accessible active reaction area, increasing electrolyte‐wettability of the carbon‐based O_2_ and CO_2_ cathodes is an effective approach. Polydopamine (PDA) coating can enhance electrolyte‐wettability of the coated carbon‐based cathode surface toward organic electrolytes, which are used in Li–O_2_ battery systems (Figure 14c).^[^ ^76^, ^158^ ^]^ As can be seen from Figure 14d, PDA‐coated carbon nanotubes (CNTs) as O_2_ cathode for Li–O_2_ batteries show higher charging and discharging capacity than those of the pristine electrode at all current densities using the LiI‐containing nonaqueous electrolyte.^[^ ^76^ ^]^ In order to improve the electrochemical properties, the CNFs are composited with an oxide catalyst (Co_3_O_4_).^[^ ^158^ ^]^ The PDA coating enables improving the electrolyte‐wettability of the CNF/Co_3_O_4_ composite surface toward a nonaqueous electrolyte. This electrolyte‐wettable increment can increase the active reaction area and catalytic activity of the O_2_ cathode. Therefore, the charging capacity of the PDA‐coated CNF/Co_3_O_4_ composite cathodes is somewhat higher than that of the uncoated CNF/Co_3_O_4_ composite pristine cathodes at current density of 1000 mA g^−1^ in the LiI‐containing nonaqueous electrolyte (Figure 14e). Carbon‐based CO_2_ cathodes for Na‐CO_2_ batteries are similar to Li–O_2_ batteries in the effect of electrolyte‐wettability of electrode on the cathode reaction. Thus, multiwall carbon nanotubes treated with ultrasonic for 2 h in tetraethylene glycol dimethyl (t‐MWCNTs) show smaller CA (13.8°) and better electrolyte‐wettability to tetraethylene glycol dimethyl ether (TEGDME) electrolyte than those of multiwall carbon nanotubes treated with ultrasonic for 2 h in ethanol, which undoubtedly benefits the cathode reaction (4Na^+^ + 3CO_2_ + 4e^−^ ↔ 2 Na_2_CO_3_ + C) in the gas/electrolyte/cathode material boundary (surface/interface) and the reduction of *R*_ct_ (Figure 14f).^[^ ^159^ ^]^ The batteries consist of a Na anode, a TEGDME electrolyte, and a t‐MWCNTs cathode, and show reversible capacity of 60 000 mAh g^−1^ at 1 A g^−1^ (≈1000 Wh kg^−1^) and run for 200 cycles with controlled capacity of 2000 mAh g^−1^ at charging voltage <3.7 V (Figure 14g). To a certain degree, the good electrolyte‐wettability of t‐MWCNTs cathode leads to reduced electrochemical polarization of the batteries and further result in high performance. However, excessive electrolyte‐wettability resulting in the formation of flooding electrode negatively impacts the energy storage performance of the carbon‐based O_2_ and CO_2_ cathode.^[^ ^160^, ^164^ ^]^ Based on simulation, the carbon‐based O_2_ cathodes have high O_2_ transfer resistance when they are fully wetted by the electrolytes, which impede the battery performance at typical electrode thickness. On the contrary, over‐dried O_2_ cathodes have poor electrochemical performance since only carbon surface that has direct contact with the electrolyte can serve as the active reaction site.^[^ ^160^ ^]^ Furthermore, the low electrolyte‐wettability leads to low ionic conductivity and high mass transfer resistance of Li^+^. So, finely constructed carbon‐based O_2_ cathodes with the mixture of electrolyte‐wettable and electrolyte‐nonwettable pores could achieve similar discharging capacity (>7 Ah g^−1^) at high current (20 A m^−2^) with electrolyte‐wettable electrodes that are fully saturated by the electrolyte (S = 100%) at low current (1 A m^−2^) (Figure 14h).

#### 3.3.2 Metal‐ and Metallic Oxide‐Based Electrodes for Alkali Metal Batteries

##### Modified Metal‐Based Electrodes by Grafting Polymer Brushes

**Figure 15:**
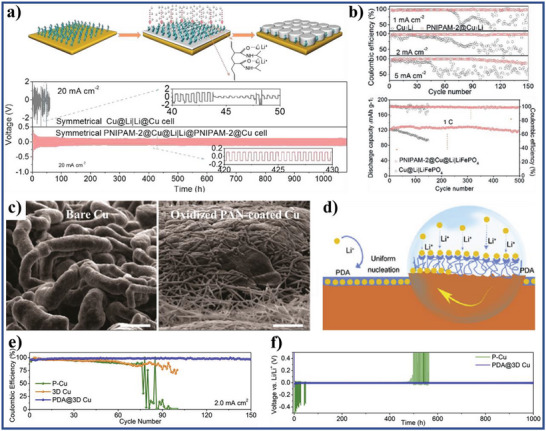
a) Homogenization of Li nucleation from the nucleation stage and normalization of Li growth can be achieved on PNIPAM polymer brushes with lithophilic functional groups modified Cu current collector and the obtained planar columnar Li anode exhibits excellent cycle stability at an ultrahigh current density of 20 mA cm^−2^ (insets: detailed voltage profiles of 40 h to 50 h, and 420 h to 430 h; the deposition capacity of Li is 5.0 mAh cm^−2^) and b) coulombic efficiency of different anodes at current densities of 1, 2, and 5 mA cm^−2^ (top) and cycling performances of PNIPAM‐2@Cu@Li||LFP and Cu@Li||LFP full batteries at the current density of 1 C (bottom). Reproduced with permission.^[^ ^165^ ^]^ Copyright 2019, Wiley‐VCH. c) Comparison of tilted‐view SEM images of Li deposition on bare Cu and oxide PAN‐coated Cu (the growth of Li is confined within the polymer fiber layer and scale bars is 5 µm). Reproduced with permission.^[^ ^30^ ^]^ Copyright 2015, American Chemical Society. d) The schematic diagram of the mechanism for the electrolyte‐wettable PDA layer guided Li metal deposition. e) Coulombic efficiency of Li deposition and stripping on the plane Cu, 3D Cu, and PDA@3D Cu at a current density of 2.0 mA cm^−2^ with a total capacity of 1.0 mAh cm^−2^ and f) voltage–time profiles of metallic Li symmetric batteries with plane Cu and PDA@3D Cu electrodes. Reproduced with permission.^[^ ^166^ ^]^ Copyright 2020, Elsevier.

The grafted Cu electrode current collector by the polymer brushes of poly(*N*‐isopropylacrylamide) (PNIPAM) exhibits much smaller CA of the electrolyte (13.4°) than that of bare Cu foil (39.7°), which indicates that the electrolyte‐wettability of PNIPAM‐2@Cu electrode current collector is enhanced significantly, moreover, the migration and diffusion of Li^+^ are further improved, because of the PNIPAM polymer brushes have abundant Li^+^‐philic functional groups of amide O (**Figure** **15**a).^[^ ^165^ ^]^ Therefore, the PNIPAM‐2@Cu current collector can induce uniform deposition of Li, which finally avoids the growth of dendrites, and exhibits excellent cycle stability and coulombic efficiency for Li metal batteries. As can be seen from Figure 15a, when the symmetrical batteries with PNIPAM‐2@Cu substrates are assembled, under the ultrahigh current density of 20 mA cm^−2^, the voltage–time curve exhibits irregular fluctuations at the beginning in the symmetrical Cu@Li||Li@Cu battery, however, the voltage–time curve shows quite stable even after 1050 h with no sign of a short circuit in the symmetrical PNIPAM‐2@Cu@Li||Li@PNIPAM‐2@Cu battery, which confirms the long‐term cycle stability of the PNIPAM‐2@Cu substrates. The coulombic efficiency of the PNIPAM‐2@Cu electrode remains 96% after 150 cycles at the 1 mA cm^−2^, while the irregular fluctuations can be observed significantly after 60 cycles for the asymmetrical battery with bare Cu foil. Along with the increase of the current density, the coulombic efficiency of PNIPAM‐2@Cu electrode remains 93%, and 90% at 2 and 5 mA cm^−2^, respectively, which could be attributed to the dendrite‐free Li deposition layer and the stable SEI formed on the interface of Li layer and the ether electrolyte (Figure 15b). The full Li metal batteries are assembled using commercial LFP and Li deposited on the PNIPAM‐2@Cu current collector as the cathode and the anode, respectively, to evaluate practical application of the PNIPAM‐2@Cu current collector. The PNIPAM‐2@Cu current collector as electrode can achieve a high discharging capacity of 124 mAh g^−1^ after 500 cycles and an excellent rate performance in the full battery (Figure 15b).

##### Polymer Surface Coating Metal‐Based Electrodes

Polymer surface coating on Cu current collector and/or Li metal electrode could adjust electrolyte‐wettability of them.^[^ ^167^ ^]^ Uniform flexible thin polymer protective layer is directly coated onto Li metal anode through spin‐coating to improve Li^+^‐philicity of the Li metal anode, because the 18‐crown‐6 applied in polymer protective layer contains O atoms that has strong Li^+^‐philicity.^[^ ^168^ ^]^ Benefitting from the high Li^+^‐philicity of 18‐crown‐6, the deposition of active Li metal during the lithiation/delithiation process is largely regulated with well‐distributed charge transfer, which suppresses Li dendrites obviously. The results of electrochemical test show that the polymer‐coated Li metal anode has a high coulombic efficiency (≈97.38% after 200 cycles) and a good cycle stability. In addition, the coated Li metal anode is applied in Li–S batteries, and performs higher capacity and better cycle stability. To investigate the significance of the O‐containing polar functional groups, the nonpolar polypropylene (PP) fiber layer and the polar oxidized PAN nanofiber layer are respectively coated on Cu current collector and/or Li metal anode as a comparison.^[^ ^30^ ^]^ The PP nanofiber‐coated Cu current collector displays a ramified growth of Li and rapid coulombic efficiency decay, as a consequence of the weak interactions between Li^+^ and the nonpolar CH_3_ groups. Conversely, the oxidized PAN nanofiber‐coated Cu current collector enables a relatively homogeneous current distribution, facilitates the uniform growth of the Li metal (Figure 15c), and shows improved cycling performance at high areal capacity (3 mAh cm^−2^) under large current density (3 mA cm^−2^) in ether‐based electrolyte. The results confirm the importance of the O‐containing polar functional groups with good electrolyte‐wettability. Compared to the complicated preparation process of the oxidized PAN nanofiber, 3D porous poly‐melamine‐formaldehyde (PMF) obtained from commercial product coating Cu current collector and/or Li metal anode is more conducive to large‐scale practical application.^[^ ^169^ ^]^ Specifically, the 3D porous PMF with many polar groups (amine and triazine) has strong electrolyte‐wettability with liquid electrolytes and thus can promote the transport of electrolyte ion, determined by density functional theory simulation. It can thus redistribute Li^+^ and reduce the ion concentration gradient caused by preferential ion flux near dendritic tips and further achieve uniform Li deposition. The galvanostatic measurements of the 3D porous PMF‐coated Cu current collector demonstrate high‐Li coulombic efficiency of 94.7% at an ultrahigh current density of 10 mA cm^−2^ after 50 cycles with low hysteresis and smooth voltage plateaus. Cycling performance of symmetrical battery using 3D porous PMF‐coated Li metal electrode in ether‐based electrolytes shows stable and smooth voltage plateaus at a high current density of 10 mA cm^−2^ with a fixed capacity of 1 mAh cm^−1^. When coupled with Li_4_Ti_5_O_12_ (LTO), the half‐battery shows enhanced rate capabilities and coulombic efficiencies.

Similar to Li metal anode, a fibrillar polyvinylidene fluoride film (f‐PVDF) with strongly polar C–F function groups coating Na metal electrode can improve electrolyte‐wettability of the electrode, which attributes to the stronger C–F polar function groups providing a better affinity to Na^+^.^[^ ^170^ ^]^ The good electrolyte‐wettability of the f‐PVDF coating layer is beneficial to Na^+^ flux and diffusion, subsequently homogenizes Na deposition and finally Na dendrite suppression. The Na–O_2_ batteries using f‐PVDF‐coated Na metal as anode successfully obtain superior electrochemical performances, such as higher rate capacities and long cycle life (up to 87 cycles) than its counterpart (only 40 cycles).

The construction of 3D electrode and current collector can inhibit the nucleation of Li dendrites and relieve the volume expansion, relied on the Sand's law, where a higher electroactive surface area enables a smaller surface current density.^[^ ^171^ ^]^ Although the performance could be improved by constructing electrode and/or current collectors with 3D porous structures to a certain extent, the inherent Li dendrite formation derived from inhomogeneous nucleation still remains inherently, especially with long‐term repeated Li deposition/stripping processes, because the inferior electrolyte‐wettability of 3D porous structures electrode and/or current collectors enables most alkali metal to plate on the out layer, pits, and crevices of 3D porous structures as soon as alkali metal ion contacts with electrons.^[^ ^155^ ^]^ The electrolyte‐wettable PDA can offer much more uniform nucleation sites and smaller nucleation overpotential for Li deposition, thus could suppress the Li dendrite growth. Therefore, the PDA thin layer‐coated 3D Cu current collector (PDA@3D Cu)^[^ ^166^ ^]^ demonstrates dendrite‐free Li deposition and stripping behavior (Figure 15d). The PDA thin layer‐coated 3D Cu current collector as electrode can remain the prominent coulombic efficiency of 96.4% after 150 cycles at the current densities of 2.0 mA cm^−2^ with 1.0 mAh cm^−2^ (Figure 15e), and run steadily over of 1000 h at the current densities of 0.5 mA cm^−2^ in Li@PDA@3D Cu||Li@PDA@3D Cu symmetric batteries (Figure 15f), as well as high cycling stability for Li||FePO_4_ full batteries.^[^ ^166^ ^]^ In addition, Tao's group adds a fluorocarbon surfactant‐1,1,2,2‐Tetrahydroperfluoro‐1‐decanol to the ether electrolyte, which could form an adsorption layer on the surface of Li metal anode before deposition reaction, because of the strong adsorption between the fluorocarbon surfactant and Li metal anode.^[^ ^172^ ^]^ The strong adsorption reduces the interfacial energy between Li metal anode and the ether electrolyte, so as to improve the wettability between Li metal anode and the ether electrolyte after adding the fluorocarbon surfactant.

##### Surface Coating of Metal‐ and Metallic Oxide‐Based Electrodes via Inorganics

**Figure 16:**
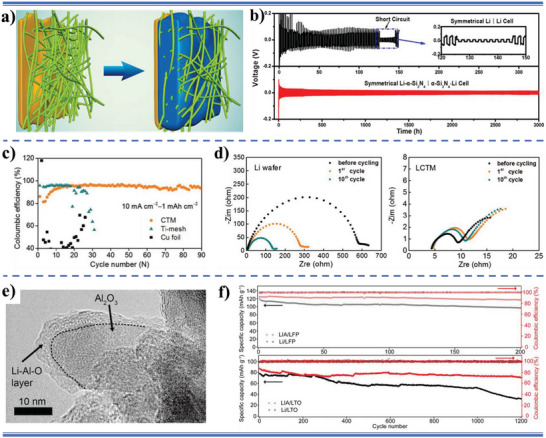
a) Schematic illustrations of Li deposition on the Cu current collector within the *α*‐Si_3_N_4_ membrane and b) voltage–time curves of Li deposition/stripping in the symmetrical Li||Li batteries (top) and the Li‐*α*‐Si_3_N_4_||*α*‐Si_3_N_4_‐Li batteries (bottom) (inset: magnified view of the voltage–time curve of the Li||Li batteries, the amount of plated Li is 1.0 mAh cm^−2^, and the current density is 1.0 mA cm^−2^ in each cycle). Reproduced with permission.^[^ ^173^ ^]^ Copyright 2018, American Chemical Society. c) Coulombic efficiency test of CTM/Li wafer, Ti‐mesh/Li wafer, and Cu foil/Li wafer at 10 mA cm^−2^ with 1 mAh cm^−2^ and d) Nyquist plots of Li wafer (left) and LCTM anode (right) for symmetrical batteries measured at 1 mA cm^−2^ with 1 mAh cm^−2^ after different cycles. Reproduced with permission.^[^ ^31^ ^]^ Copyright 2019, Wiley‐VCH. e) High‐resolution TEM (HRTEM) image of LIA anode after stripping Li completely from the skeleton; and f) galvanostatic cycling performance of LIA||LFP and bare Li||LFP full batteries at a rate of 1 C (top), and LIA||LTO and bare Li||LTO full batteries at a rate of 4 C (bottom). Reproduced with permission.^[^ ^77^ ^]^ Copyright 2018, Wiley‐VCH.

Except for polymer surface coating, inorganics surface coating could also improve electrolyte‐wettability of metal‐based and metallic oxide‐based electrode. A porous *α*‐Si_3_N_4_ fibrous membrane features abundant polar covalent bonds on its surfaces to interact strongly with Li^+^. Thus, porous *α*‐Si_3_N_4_ fibrous membranes coating Cu or Li electrode enables good electrolyte‐wettability, Li dendrite suppression, and finally remarkable electrochemical performance (**Figure** **16**a).^[^ ^173^ ^]^ The coulombic efficiency of the *α*‐Si_3_N_4_‐membrane‐coated Cu (Cu‐*α*‐Si_3_N_4_) electrode remains nearly constant at a high level of ≈90% at 0.5, 1, and 2 mA cm^−2^ after 100 cycles, while the coulombic efficiency of the bare Cu significantly decays. Symmetric Li‐*α*‐Si_3_N_4_||*α*‐Si_3_N_4_‐Li batteries, assembled using two identical *α*‐Si_3_N_4_‐membrane‐coated Li metal sheets as the electrodes can continuously operate for more than 3000 h without any sign of a short circuit. Inversely, the typical short‐circuiting behavior is found for the symmetric bare Li||Li batteries after only 150 h due to dendrite growth (Figure 16b).

Li^+^‐philic graphitic carbon layer and Li^+^‐philic nanoflower‐like CuO coating Ti‐mesh electrode current collector could lower the interfacial energy of Li deposition, enhance electrolyte‐wettability of the anode, and improve the distribution of the electric field to lower the local current densities, which would finally induce Li uniform deposition.^[^ ^31^ ^]^ As a result, the nanoflower‐like CuO‐coated Ti‐mesh (CTM) as current collector of half‐battery maintains a high CE of 94.2% over 90 cycles even at 10 mA cm^−2^ (Figure 16c). The circular disks of CTM are pressed on the same diameter Li wafer by battery sealer with constant mechanical pressure of 800 psi at 25 °C in a glove box filled with Ar to obtain Li/CuO@Ti‐mesh (LCTM) composite anode. The LCTM anode and Li wafer are assembled into symmetrical batteries using ether‐based electrolyte 1 m lithium bis(trifluoromethanesulfonyl)imide) (LiTFSI) in DOL and DME (1:1, V:V). The batteries with LCTM anode exhibit stable voltage profiles and lower overpotential than batteries with Li wafer. Through performing EIS, the LCTM batteries show a lower resistance of 10 than 580 Ω of Li wafer batteries before cycling (Figure 16d). Considering that metallic oxide (CuO, Al_2_O_3_) has low energy barriers for electrolyte wetting and nucleation, when acted as an electrode, it can induce the uniform nucleation and growth of Li metal.^[^ ^31^, ^77^ ^]^ The surface of Al_2_O_3_ particles is coated by Li^+^‐philic Li–Al–O interface layer formed by simply reacting molten Li with Al_2_O_3_ particles (Figure 16e), which can effectively redistribute Li^+^, reduce the ion concentration gradient near surface protrusion, and regulate their deposition behavior, thus further suppressing the initiation of dendrite growth.^[^ ^77^ ^]^ From galvanostatic measurements, symmetric batteries using the Li–Al–O layer coated 3D Al_2_O_3_ (LIA) electrode can conduct under an ultrahigh current density of 8 mA cm^−2^ over 480 cycles with capacity fixed at 1 mAh cm^−2^. When used in full batteries, it enhances the capacity retention of Li||LFP from 78.4% to 93.6% after 200 cycles at 1 C and enables stable long‐term cyclability of Li||LTO for over 1200 cycles at 4 C (Figure 16f).

#### 3.3.3 Zn Metal Anode for Zn Metal Batteries

##### Polymer Coating Zn Metal Anode

**Figure 17:**
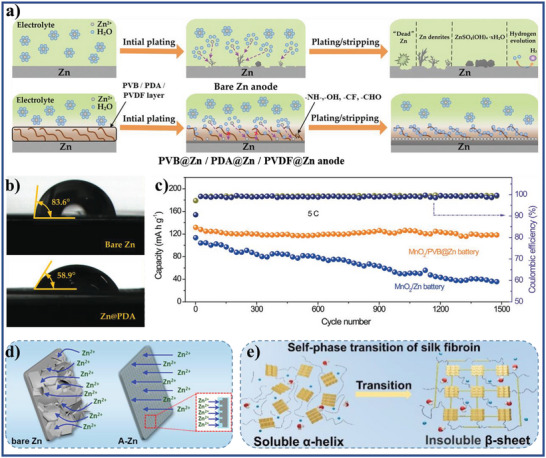
a) Schematic illustration of morphology evolution for the bare Zn–Zn batteries, the PVB@Zn‐PVB@Zn, PDA@Zn‐PDA@Zn, and PVDF@Zn‐PVDF@Zn batteries during repeated cycles of stripping/plating, and b) in situ CA measurements of bare Zn and PDA@Zn foil, respectively. Reproduced with permission.^[^ ^32^ ^]^ Copyright 2022, Wiley‐VCH. c) Long‐term cycling stability of MnO_2_/Zn and MnO_2_/PVB@Zn batteries at 5 C with the corresponding CEs. Reproduced with permission.^[^ ^78^ ^]^ Copyright 2020, Wiley‐VCH. d) Schematic illustrations showing Zn deposition behavior on the bare Zn and APTES coated Zn surface. Reproduced with permission.^[^ ^82^ ^]^ Copyright 2022, American Chemical Society. e) Schematic illustration of the self‐phase transition of silk fibroin molecule layer. Reproduced with permission.^[^ ^80^ ^]^ Copyright 2022, Elsevier.

Zn metal batteries, as promising aqueous rechargeable batteries, are attributed to high theoretical capacity (gravimetric capacity of 820 mAh g^−1^ and volumetric capacity of 5855 mAh cm^−3^) and a low reduction potential (−0.76 V vs standard hydrogen electrode (SHE)).^[^ ^151^, ^152^ ^]^ Thermodynamically, increasing electrolyte‐wettability of Zn anode surface could reduce the interfacial energy between the Zn anode and the aqueous electrolyte, promote easier access of Zn^2+^ to Zn metal anode surface, and render the uniform distribution of electrolyte ions on the Zn surface, contributing to the formation of homogeneous nucleation and deposition, which plays a crucial role in the final Zn uniform deposition (**Figure** **17**a).^[^ ^78^, ^174^, ^175^ ^]^ Some polymer films with rich polar functional groups such as poly(vinyl butyral) (PVB), PDA, poly(vinylidene difluoride) (PVDF) generally exhibit significant electrolyte‐wettability, because the polarity of those polar functional groups is close to the polarity of water and has more negative binding energy with Zn^2+^ (—OH, —NH_2_, and, —COH are −235.45, −241.30, and −295.74 kcal mol^−1^).^[^ ^32^, ^79^, ^80^ ^]^ Thus, electrolyte‐wettability of Zn metal anode toward aqueous electrolytes could be enhanced by coating the electrolyte‐wettable polymer films on surface of the Zn anode using facile spin‐coating^[^ ^78^ ^]^ and/or scrape‐coating (Figure 17a,b).^[^ ^32^ ^]^ The PVB‐coated Zn (PVB@Zn) anode delivers an extended deposition and stripping cycling life of 2200 h in symmetric batteries at the current density of 0.5 mA cm^−2^.^[^ ^78^ ^]^ Coupling with LFP cathode, the full hybrid LFP||PVB@Zn batteries get an initial CE of 96.3% (capacity of ≈153.5 mAh g^−1^), higher than that of the batteries with bare Zn electrode (an initial CE of 93.1% with a capacity of ≈149.2 mAh g^−1^). After 1500 cycles, the assembled MnO_2_||PVB@Zn batteries using MnO_2_ as cathode replacing LFP show much higher capacity retention of 86.6% than that of the MnO_2_||bare Zn batteries (31.8%) (Figure 17c).

In order to get that the electrolyte‐wettable polymer film could be conformally and firmly coated on the surface of Zn metal anode, (3‐aminopropyl)triethoxysilane (APTES), which is commonly used to form a foundation layer and increase adhesion, is one of the best choices as a coating layer to improve the surface electrolyte‐wettability of Zn metal anode, because APTES not only has hydrophilic amine groups but also silanol groups that could react with the hydroxyl functional group on Zn metal anode surface. APTES film forms a thin (≈500 nm), hydrophilic, and highly uniform coating layer by covalently attaching to hydroxylated Zn surface, promoting electrolyte‐wettability of the Zn metal anode, allowing more even Zn^2+^ flux across the surface of Zn, and resulting in homogeneous zinc plating and nucleation without dendrite growth (Figure 17d).^[^ ^82^ ^]^ Zhu and co‐workers propose eco‐friendly silk fibroin as an effective electrolyte additive and disclose that the silk fibroin molecules would first adsorb on Zn metal anode surface (due to the strong adsorption interaction between silk fibroin and Zn metal) and then undergo a self‐phase transition process to transform form soluble *α*‐helices to insoluble *β*‐sheets (Figure 17e), thereby in situ yielding a polymer coating layer.^[^ ^80^ ^]^ The rich polar groups in silk fibroin coating layer confer Zn metal anode surface with excellent electrolyte‐wettability, which could effectively improve the diffusion kinetics of Zn^2+^ and evenly distribute the nucleation sites, thus suppressing the dendtritic growth. Besides, *γ*‐butyrolactone as an additive of aqueous electrolytes also improves electrolyte‐wettability of Zn metal anode, and its principle is similar to that of the silk fibroin additive.^[^ ^176^ ^]^

##### Zincophilic Metals and Inorganics Coating Zn Metal Anode

**Figure 18:**
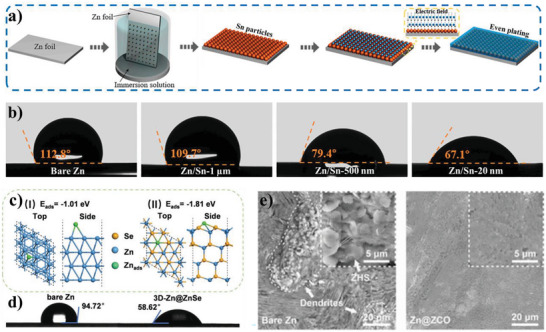
a) Schematic illustration of preparing Zn electrodes with Sn particles and Zn deposition on Zn/Sn‐20 nm electrodes. b) The CA on bare Zn, Zn/Sn‐1 µm, Zn/Sn‐500 nm, and Zn/Sn‐20 nm. Reproduced with permission.^[^ ^177^ ^]^ Copyright 2022, Elsevier. c) DFT calculations of the adsorption energy of Zn atoms on the bare Zn and the ZnSe coating layer, respectively. d) CA of 2 m ZnSO_4_ droplets on these two substrates. Reproduced with permission.^[^ ^178^ ^]^ Copyright 2022, Wiley‐VCH. e) SEM images of bare Zn and Zn@ZCO anodes after 50 cycles. Reproduced with permission.^[^ ^55^ ^]^ Copyright 2022, Wiley‐VCH.

Numerous efforts have been put to coat zincophilic metals and inorganics on Zn metal anode to improve the electrolyte‐wettability of the Zn metal anode surface, which is because the strong interfacial interaction between the zincophilic metal and Zn^2+^ drag the electrolyte containing the Zn^2+^ to wet the surface of the Zn metal anode surface.^[^ ^84^ ^]^ Zincophilic metals include Sn metal,^[^ ^177^ ^]^ eutectic ZnAl alloy,^[^ ^179^ ^]^ Cu–Zn alloy,^[^ ^83^ ^]^ and so on. The in situ formulation of coating layer by ultrafine and homogeneous Sn particles on the surface of the Zn metal anode with a facile impregnation method is reported by Wang et al. (**Figure** **18**a). The rationally designed coating layer featuring an improved electrolyte‐wettability markedly enhances uniform electric field, exposes more active sites, promotes Zn^2+^ transport kinetics, thereby lowering the nucleation energy barrier and suppressing the growth of Zn dendrites (Figure 18a).^[^ ^177^ ^]^ The detailed comparisons of the electrolyte‐wettability of various sized Sn coated Zn metal anodes show the improvement in electrolyte‐wettability of the surface with large Sn particles is limited (the CA of 112.8° and 109.7° for bare Zn and Zn/Sn‐1 µm) and Zn/Sn‐20 nm is the most effective for electrolyte wetting, with a CA of 67.1° compared with 79.4° for Zn/Sn‐500 nm (Figure 18b) (Zn/Sn‐20 nm, Zn/Sn‐500 nm, and Zn/Sn‐1 µm indicate that the size of Sn particles on the surface of Sn coated Zn metal anode is 20, 500, and 1 µm). This is because the zincophilicity of Sn could improve the electrolyte‐wettability of Zn metal anode, and the increase of surface energy of ultrafine Sn particles could further increase the electrolyte‐wettability of Zn metal anode.

Apart from zincophilic metal, some inorganics are coated on Zn metal anode surface to improve its electrolyte‐wettability, such as ZnSe,^[^ ^84^, ^178^ ^]^ SiN,^[^ ^85^ ^]^ Si_3_N_4_,^[^ ^180^ ^]^ zinc oxalate (ZnC_2_O_4_),^[^ ^55^ ^]^ and so on, which is ascribed to the strong adsorption between Zn^2+^ and nonmetallic element from the inorganics as well as Zn^2+^ and N‐ and O‐rich functional groups on the surface of the inorganics. DFT calculation model is utilized to investigate the adsorption energy of bare Zn anode and ZnSe with Zn^2+^ (Figure 18c).^[^ ^178^ ^]^ The adsorption energy between the bare Zn and Zn^2+^ is calculated to be −1.01 eV. In contrary, the ZnSe coating layer shows a higher adsorption energy of −1.81 eV. The results of the CA measurement also demonstrate that the ZnSe coating layer has a better wettability for ZnSO_4_ electrolyte (Figure 18d). Electronegative carbonyl oxygen of the ZnC_2_O_4_ not only exhibits strong nucleophilic ability when coordinated with the metal ion, but also exhibits sufficient hydrophilicity.^[^ ^55^ ^]^ Inspired by this synergy, coating the carbonyl‐containing ZnC_2_O_4_ layer on Zn metal anode surface (Zn@ZCO) improves electrolyte‐wettability of Zn metal anode, facilitates electroyte/anode interface connection and Zn^2+^ transport kinetics, thus homogenizing the deposition of Zn metal (Figure 18e).

##### Manipulating Structure of Zn Metal Anode

**Figure 19:**
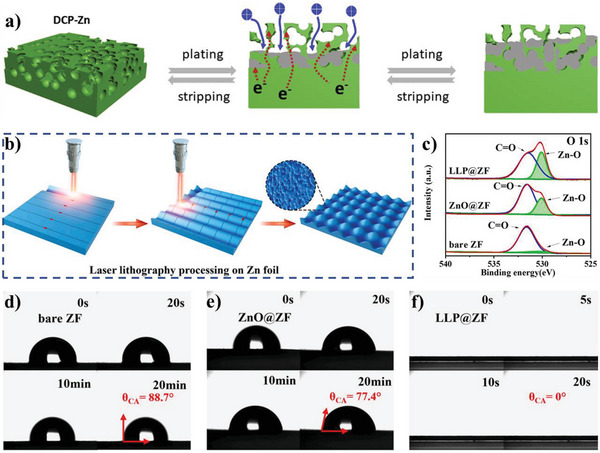
a) Schematic illustration of the 3D porous structure and dual‐channel skeletons endowing the DCP‐Zn with suppressed dendrite growth and promoted kinetics. Reproduced with permission.^[^ ^86^ ^]^ Copyright 2020, Elsevier. b) Schematic illustrating the top‐down fabrication of the LLP@ZF by nanosecond laser lithography. c) High‐resolution XPS O 1s spectra of LLP@ZF, ZnO coated ZF (ZnO@ZF), and bare ZF. d‐f) Optical images of the 2 m ZnSO_4_ electrolyte‐electrode CA, comparing LLP@ZF versus ZnO@ZF and bare ZF. Reproduced with permission.^[^ ^181^ ^]^ Copyright 2022, Elsevier.

According to classical Wenzel theory, when the electrode is intrinsically electrolyte‐wettable (*θ* < 90°), an increase of surface roughness has an amplification effect for surface electrolyte‐wettability.^[^ ^4^ ^]^ Therefore, in addition to lowering the local current density, accelerating the electrons transfer, and buffering the volume variation, the dual‐channel 3D porous Zn (DCP‐Zn) has better electrolyte‐wettability by aqueous electrolyte than pristine Zn foil due to its higher roughness. The DCP‐Zn electrode favors the electrolyte penetration and ion transports to the electrode/electrolyte interfaces and displays dentrite‐free Zn deposition and finally excellent electrochemical performance (longer cycling lifetime of 1400 h under 0.1 mAh cm^−2^ at 0.5 mA cm^−2^ (**Figure** **19**a) and 200 h under 10 mAh cm^−2^ at 5 mA cm^−2^ in symmetric batteries).^[^ ^86^ ^]^ An established metallurgical surface finishing method based on laser processing adapts to fabricate a periodic concave–convex patterned Zn foil anode (LLP@ZF) from commercial Zn foils (ZF) (Figure 19b).^[^ ^181^ ^]^ The laser patterning process introduces ZnO layer on the Zn surface (Figure 19c), while the surface roughness is significantly enhanced (roughness 16.5 times planar). The introducing ZnO layer improves intrinsic wettability (Figure 19d,e), because the surface energy of ZnO is more than twice as high as that of bare Zn. Then, the major enhancement in electrolyte‐wettability of LLP@ZF (Figure 19f) is mainly attributed to its roughness that amplifies the benefit from the oxide.

In metal‐based batteries, the dendrites of active metals formed during anodic deposition and striping are the most important problem, which leads to the decrease of energy storage and safety performance of the batteries. Excellent electrolyte‐wettability of electrode could lower the interfacial energy of electrode/electrolyte interface, facilitate migration and diffusion of metal ions to reduce the local current densities of electrode surface, redistribute metal ions, and reduce the ion concentration gradient caused by preferential ion flux near dendritic tips, as well as form thin and stable SEI film, which renders uniform nucleation site and dendrite‐free active metal deposition. Thus, improving electrolyte‐wettability of electrode is commonly beneficial to increase coulombic efficient and rate capability of the metal‐based half‐batteries, reduce nucleation overpotential and hysteresis of the voltage plateaus of metal deposition process, augment energy density, power density, cycling stability, and safety performance of the metal‐based batteries that be assembled by the electrode, as well as decrease interfacial impedance and *R*_ct_. For cathode in metal‐based batteries, such as carbon‐based O_2_ cathode in metal–O_2_ batteries and CO_2_ cathode in metal–CO_2_ batteries, the cathode with well electrolyte‐wettability could also ensure high accessible active reaction area, and fast ion transport and diffusion in the cathode, which improves charging and discharging capacity, rate capacity, and *R*_ct_ of the batteries, while excessive electrolyte‐wettability resulting in the formation of flooding electrode negatively impacts the energy storage performance of the cathodes.

## 4 Electrolyte‐Wettability of Electrode Materials in Electrochemical Energy Conversion Systems

### 4.1 Electrolyte‐Wettability in Fuel Cells

**Figure 20:**
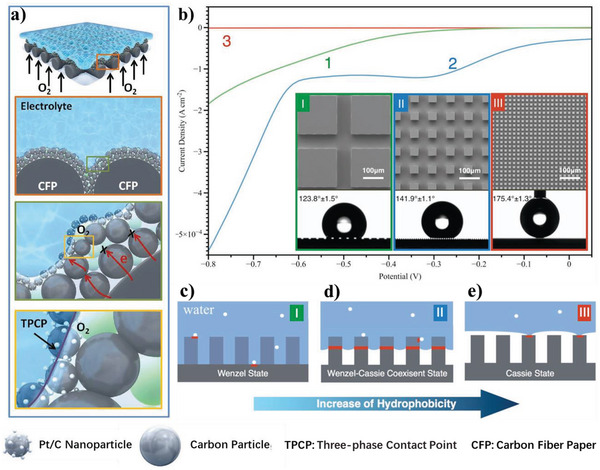
a) Schematic illustration of the commercial air electrode with an additional MPL located between CFP and Pt/C catalyst (Pt/C‐MPL‐CFP) and a three‐phase contact point (TPCP) for oxygen (gas), electrolyte (liquid), and catalyst (solid) under electrolyte. Reproduced with permission.^[^ ^182^ ^]^ Copyright 2016, Wiley‐VCH. b) The linear sweep voltammetry curves of ORR reaction on three platinum‐coated samples (the inset shows the SEM images of the three conductive columnar structures and the water CAs of the three samples). For the underwater system, the typical three wetting state of a hydrophobic surface can be labeled as the nature of gas–liquid–solid interface, as schematically shown in c) underwater Wenzel state, d) underwater Wenzel–Cassie coexistent state, and e) underwater Cassie state (the schematic diagram of three‐phase interface is denoted by the red color). Reproduced with permission.^[^ ^183^ ^]^ Copyright 2017, Wiley‐VCH.

Fuel cells continue to be at the forefront of alternative energy technologies to current fossil‐based energy generation technologies.^[^ ^184^ ^]^ In fuel cells, electricity is produced mainly from clean energy sources (including H_2_, alcohols, and air) achieving high‐power density and efficiency with low emission levels. Just like supercapacitors and batteries, fuel cells consist of an anode, where oxidation occurs, a cathode, where reduction occurs, and an electrolyte (generally refer to aqueous electrolyte), where ions carry the current between the electrodes.^[^ ^20^ ^]^ Thus, sufficient electrolyte‐wettability of electrode is required to maintain close contact between catalytic active sites and electrolyte and the transport of proton in the inner channel of the electrode.^[^ ^182^ ^]^ However, gas–liquid–solid three‐phase reaction system (**Figure** **20**a) in fuel cells is different from solid–liquid two‐phase reaction system in supercapacitors and some batteries.^[^ ^182^ ^]^ Excessive electrolyte‐wettability can block the pores of the catalyst layer and porous transport layer of the electrode, impeding the transport of reactant gas to the active sites, thus resulting in the formation of flooding electrode and mass transport losses limiting the power density.^[^ ^37^, ^185^ ^]^ Therefore, designing optimal balance of electrolyte‐wettability for electrode is critical for high power performance and durability for fuel cells. The electrode applied in fuel cells is generally composed of catalyst layer, gas diffusion layer, and polar plate. Thus, electrolyte‐wettability of the electrode could be adjusted by optimizing the electrolyte‐wettability of catalyst layer, gas diffusion layer, and polar plate, respectively.

#### 4.1.1 Controlling Electrolyte‐Wettability of Catalyst Layer

When a fuel cell is operated, oxygen in the flow channels of polar plate diffuses through the pores of the gas diffusion layer to reach the cathode catalyst layer, where the oxygen reduction reaction (ORR) that is at the heart of the fuel cell takes place.^[^ ^186^ ^]^ The ORR, a typical gas‐consuming process happening at gas–liquid–solid three‐phase interface, is viewed as a major limiting factor to enhance the efficiency of fuel cells.^[^ ^183^ ^]^ Considering the rather low solubility and diffusion rate of O_2_ in electrolyte solutions, practical ORR catalyst layer in fuel cells is usually assembled with one side (the wet side) contacted with the electrolyte and the other side (the dry side) exposed to air. In the electrocatalytic ORR, the dry side maintains steady gas reservoir to guarantee sufficient O_2_ supply, while the wet side provides sufficient pathway for electrolyte percolation.^[^ ^187^ ^]^ Therefore, controlling the electrolyte‐wettability of the catalyst layer plays an important role in stabilizing the gas–liquid–solid three‐phase interface.

Currents strategies to modify the surface wettability of electrocatalysts mainly rely on morphology control and surface coating while the content and activity of loaded catalysts are usually decreased accordingly. Thus, Wang et al. design three platinum‐plated sample with different surface texture and water CA, and investigate their effect on the ORR performance of the electrodes (Figure 20b).^[^ ^183^ ^]^ The hydrophobicity gradually increases with increases of the roughness due to smaller size of silicon pillars. The sample III has the strongest hydrophobicity and O_2_ absorption capacity, which is generally considered to be a favorable factor for ORR. However, the sample III is the worst for the ORR catalytic performance. Noted that sample II has a balanced adhesion to electrolyte and O_2_, and the ORR catalytic performance is the highest. Thus, it is suggested that the superhydrophobic catalyst layer with particularly underwater Wenzel–Cassie coexistent state (Figure 20d) is the essence for maximizing and stabilizing the underwater gas–liquid–solid three phase interface, by which the O_2_ diffusion and ionic transportation on the electrode could be drastically facilitated, as well as the rate of the reaction with gas as the reactant. To be noticed, enlarging either liquid–solid (underwater Wenzel state (Figure 20c)) or gas–solid (underwater Cassie state (Figure 20e)) two phase interface will drastically retard the reaction proceeding. The gas–liquid–solid three phase interface could also be stabilized by coating hydrophobic polymer on catalyst layer.^[^ ^188^ ^]^ However, the resultant may lead to an unwanted barrier that block the direct contact between O_2_ and catalyst. The Janus electrodes with asymmetric wettability are made based on N‐doped CNTs grown on the carbon fiber paper, followed by coating singe‐side with PTFE solution via a finely tuned finite‐depth capillarity deposition process.^[^ ^187^ ^]^ The PTFE coating imparts the modified side with hydrophobicity to guarantee sufficient O_2_ supply, while the other side without PTFE coating preserves its original hydrophilicity to provide efficient ionic transportation and the direct contact between O_2_ and catalyst. The optimized Janus electrode exhibits superior ORR activity with a potential of 0.5 V to reach current density of 22.5 mA cm^−2^ compared to merely hydrophobicity (11.2 mA cm^−2^@0.5 V) or hydrophilicity (3.3 mA cm^−2^@0.5 V) electrodes, which have the shortage in either ion or oxygen supply. In addition, the polar atom doping may also tune electrolyte‐wettability of metal‐free carbon‐based catalysts and carbon supported noble metal catalysts to enhance their ORR performance except for inducing defects, charge density, and electronic‐structure modulations for contributing ORR performance,^[^ ^95^, ^189^, ^190^ ^]^ despite it is little mentioned in previous reports.

#### 4.1.2 Controlling Electrolyte‐Wettability of Gas Diffusion Layer

The role of a gas diffusion layer is to allow gaseous reactants to move to the catalyst layer and provide a water pathway from the catalyst layer to the flow channel of the bipolar plates. To avoid excessive water accumulation in the gas diffusion layer, usually a hydrophobic material (PTFE) coating is applied, which enhances the water removal capacity of the gas diffusion layer.^[^ ^192^, ^193^ ^]^ However, excessive coating of PTFE leads to a lower water content of the gas diffusion layer and increases the hydraulic pressure for driving the liquid water flow through the pores.

**Figure 21:**
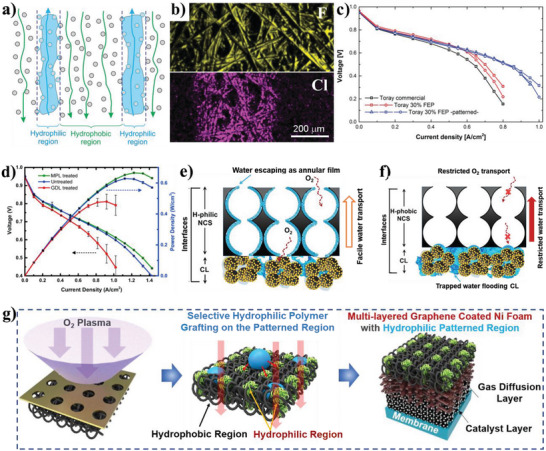
a) Illustration of the relationship between the pathways for liquid water (blue) and for reactant gases (green) in gas diffusion layer with patterned hydrophilicity. b) EDX elemental mapping of a modified region of 500 µm showing two elements: F (representative of coating), and Cl (representative of the grafted modification and ion exchange), and c) polarization curves at 50 °C, 100/30% RH anode/cathode of the following three materials. Toray commercial, Toray 30% FEP (Toray TPG‐H‐060 coated in‐house with 30%, FEP), Toray 30% FEP‐patterned‐ (Toray TPG‐H‐060 coated in‐house with 30% FEP and pattern‐grafted with NVF). Reproduced with permission.^[^ ^38^ ^]^ Copyright 2015, Wiley‐VCH. d) Polarization and power curves for the single cell with different membrane‐electrode assemblies under wet conditions (60 °C, 100% RH). Reproduced with permission.^[^ ^185^ ^]^ Copyright 2018, Elsevier. e) Depiction of gas and water transport through the MPL with a focus on the catalyst layer‐MPL interface at dry conditions (≤80% RH) e) H‐philic NCS and f) H‐phobic NCS. Reproduced with permission.^[^ ^191^ ^]^ Copyright 2020, American Chemical Society. g) Schematic illustrations of preparation of MLG‐coated Ni foam with patterned wettability. Reproduced with permission.^[^ ^192^ ^]^ Copyright 2019, American Chemical Society.

Recently, using the concept of patterned electrolyte‐wettability, the localized electrolyte‐wettability on the previous electrolyte‐phobic gas diffusion layer of electrode is induced by radiation grafting electrolyte‐wettable polymer (PNVF) to engineer the patterned electrolyte‐wettability within the gas diffusion layer.^[^ ^37^, ^38^ ^]^ The gas diffusion layer with patterned electrolyte‐wettability provides some artificially creating pathways for proton transport and water removal (hydrophilic region), while leaving the remaining region of electrolyte‐phobicity (hydrophobic region) for improving mass or reactant gas transport, as shown in **Figure** **21**a. The electrolyte‐wettable stripes on the gas diffusion layer with patterned electrolyte‐wettability could be found by elemental mapping with energy dispersive X‐ray (EDX) spectroscopy, which is based on the electrolyte‐wettable area having the presence of chlorine (Cl) after forming the quaternary ammonium salt that is conducive to improve electrolyte‐wettability (Figure 21b).^[^ ^38^ ^]^ By evaluating the electrochemical performance using the modified electrode with patterned electrolyte‐wettability, a substantial performance increase is observed when comparing with baseline materials, especially at high current density (*i* > 0.6 A cm^−2^), where mass transport is limited (Figure 21c). Other attempts of altering the electrolyte‐wettability of gas diffusion layer in the patents include the exposure of gas diffusion layer to plasma sources (SiH_4_, O_2_ or both) and the addition of electrolyte‐wettable metal oxide particles for developing an electrolyte‐wettable surface on top of electrolyte‐phobic substrate.^[^ ^194^, ^195^ ^]^ However, for practical applications, the modification methods must be simple, scalable, and cost‐effective. The state‐of‐the‐art design of the porous carbon layer for the electrodes within the fuel cell consists of a bilayer structure with a gas diffusion layer and a microporous layer (MPL).^[^ ^196^ ^]^ The O_2_ plasma modification method generating higher concentration of O‐containing functional groups represents a fast, environmentally‐friendly, and scalable approach for controlling the electrolyte‐wettability of gas diffusion layer and MPL.^[^ ^185^, ^197^ ^]^ It could be found that the modified MPL by hydrophilic plasma treatment shows better fuel cell performance, oppositely, the modified gas diffusion layer does not provide an advantageous effect on the fuel cell performance (Figure 21d). This could be attributed to the electrolyte‐wettable increase in the MPL where it could reduce the resistance of water flow through the micropores, minimizing water accumulation at the catalyst layer/MPL interface.^[^ ^185^ ^]^ Conversely, the modified gas diffusion layer having an inferior performance from the beginning of the test and current densities higher than 0.7 A cm^−2^ could be attributed to a kinetic limitation where the water present in the humidified gas does not efficiently reach the surface of the catalyst layer.

A nanoporous carbon scaffold (NCS) with controlled wettability is presented as a model MPL. The electrolyte‐wettability of the internal surface of the NCS is tuned using in situ diazonium method to anchor hydrophilic (phenyl sulphonate‐PhSO_3_H) and hydrophobic (2,3,4,5,6‐pentafluorophenyl‐PhF_5_) functional groups.^[^ ^191^ ^]^ The functionalized electrolyte‐philic and electrolyte‐phobic NCS are denoted as H‐philic NCS and H‐phobic NCS, respectively. Figure 21e,f illustrates the water and oxygen transport in the H‐philic and H‐phobic NCS adjacent to the catalyst layer, respectively. It is suggested that unlike spherical droplets on an electrolyte‐phobic NCS and a conventional MPL, liquid water tends to form an annular film within the pores of the H‐philic NCS, which leaves the pores open for oxygen transport. Moreover, the high electrolyte‐philic surface of the NCS allows liquid water to redistribute, which improves the water capacity of the catalyst layer. Due to the two combined effects of annular water transport and a high electrolyte‐philic surface area, the H‐philic NCS in 60% and 80% RH (relative humidty) shows the least O_2_ transport resistance and has the highest transition limiting current.

#### 4.1.3 Controlling Electrolyte‐Wettability of Flow Distributor

Bipolar plates are the most important component of fuel cell because they function as current conductors between the cells and as a passage for supplying reactant gas and removing water.^[^ ^192^ ^]^ To distribute reactant gas to catalyst layer and transport product water, flow channels are usually machined on the surfaces of the bipolar plates. However, it has issues associated with the flow channels, such as high manufacturing cost, heavy weight, and difficulty in processing various channels. As an alternative, a metal foam as a porous flow distributor has been introduced in bipolar plates owing to their open‐pore structure (porosity > 90%), lightness, and excellent electrical and thermal conductivity. Because the flow distributor has to facilitate the transport of reactant gas and water removal, the metal foam needs to be electrolyte‐phobic on surface.^[^ ^198^ ^]^ Electrolyte‐phobic modifications on the metal foams mainly focus on coating superhydrophobic PTFE on the surface to enhance water removal and corrosion–protection as well.^[^ ^199^ ^]^ Yoo and co‐workers report a modified Ni foam as an effective 3D porous flow distributor by multilayered graphene (MLG) coating followed by hydrophilic polymer grafting on the prepatterned region (Figure 21g).^[^ ^192^ ^]^ The MLG is completely coated on the entire region of the 3D structured Ni foam surface by a gas phase deposition technique. The MLG‐coated Ni foam reduces the interfacial contact resistance between the gas diffusion layer and Ni foam by surface passivation of the oxide layer on the Ni foam and changes the surface wettability of the Ni foam from electrolyte‐philic to electrolyte‐phobic. Furthermore, the formation of a patterned electrolyte‐philic region in the electrolyte‐phobic MLG‐coated Ni foam provides water drainage pathway through an electrolyte‐philic region, while the electrolyte‐phobic region served as a gas pathway. By utilizing the patterned wettability to separate gas/water passages in the 3D porous metal foam flow distributor, the electrochemical performance of the fuel cell is significantly improved with high power performance of ≈920 mW cm^−2^ and low ohmic resistance of ≈0.052 Ω cm^2^.

### 4.2 Electrolyte‐Wettability in Electrochemical Water Splitting Systems

The OER and HER are of paramount importance for electrochemical water splitting systems.^[^ ^200^ ^]^ In general, a typical OER and/or HER process involves H_2_O adsorption, activation, and O_2_ and/or H_2_ desorption.^[^ ^54^ ^]^ Thus, an electrolyte‐wettable surface is desirable for the electrode been used in OER and HER because it can facilitate the initial adsorption of water (reactants) on the electrode material surface and promote detachment of the evolved H_2_ or O_2_ bubbles from the electrode material surface, which is beneficial to the HER or OER catalytic kinetics.^[^ ^34^, ^35^, ^201^ ^]^ According to the measures for improving the electrolyte‐wettability of electrode materials analyzed in Section 2.5, three main approaches could be applied to enhance the electrolyte‐wettability of the electrocatalyst used in HER and OER, including surface coating, structural designing, and surface functionalization.

#### 4.2.1 Surface Coating for Improving Electrolyte‐Wettability of Electrocatalyst

**Figure 22:**
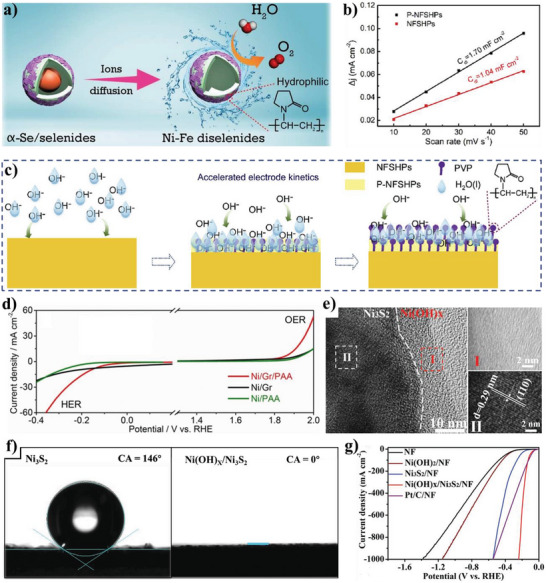
a) Schematic illustration of improving electrolyte‐wettability of Ni–Fe diselenide catalyst by coating hydrophilic PVP. b) Plots of the current density (at 1.15 V) versus the scan rate of NFSHPs and P‐NFSHPs, and c) schematic mechanism diagram of the functional PVP on Ni–Fe diselenide hollow nanoparticles toward OER. Reproduced with permission.^[^ ^202^ ^]^ Copyright 2018, Elsevier. d) The *iR*‐corrected the linear sweep voltammetry (LSV) curves of the Ni/Gr/PAA, Ni/Gr, and Ni/PAA electrodes for OER and HER measured in O_2_/N_2_ saturated 1.0 m phosphate buffer saline (PBS) solution (pH 7.0), respectively. Reproduced with permission.^[^ ^188^ ^]^ Copyright 2018, Wiley‐VCH. e) HRTEM images of Ni(OH)*_x_*/Ni_3_S_2_/NF, f) CA of Ni_3_S_2_ and Ni(OH)*_x_*/Ni_3_S_2_, and g) LSV curves of NF, Ni(OH)_2_/NF, Ni_3_S_2_/NF, Ni(OH)*_x_*/Ni_3_S_2_/NF, and Pt/C/NF. Reproduced with permission.^[^ ^203^ ^]^ Copyright 2022, Wiley‐VCH.

The surface coating is broadly used to modify the electrolyte‐wettability of electrode in early years. The interfacial energy between electrodes and electrolytes could be directly tuned by coating controllable chemical compositions.^[^ ^204^ ^]^ The highly hydrophilic PVP is directly coated on the surface of Ni–Fe diselenide nanoparticles electrocatalyst by a one‐pot hydrothermal method to reduce the interfacial energy between the electrocatalyst and electrolyte and improve electrolyte‐wettability of the electrocatalyst (**Figure** **22**a).^[^ ^202^ ^]^ The modified Ni–Fe diselenide hollow nanoparticles by PVP (P‐NFSHPs) with electrolyte‐wettable feature exhibits enlarged catalytic activity with the *C*_dl_ of 1.7 mF cm^−2^ (Figure 22b) and enhanced OER performance with a low overpotential of 255 mV (vs reversible hydrogen electrode) at 10 mA cm^−2^, a low Tafel slope of 56 mV dec^−1^, and decent long‐term stability. The reason is that the lactam group with strong polarity in PVP could interact with polar H_2_O molecules, which facilitates the adsorption/transfer efficiency of the ion species and eventually accelerates the kinetics of OER (Figure 22c). Zhang and co‐workers report that an adlayer of hydrophilic polymer PAA is coated onto the outer surface of the N‐doped graphene monolith (Gr) loaded onto Ni current collector (Ni/Gr), resulting in an electrolyte‐wettable surface of the as obtained electrode (Ni/Gr/PAA) with a CA of 0°.^[^ ^188^ ^]^ The Ni/Gr/PAA electrode exhibits a significant decrease in overpotential for OER at 10 mA cm^−2^ from 730 mV on Ni/Gr to 652 mV on Ni/Gr/PAA, and a similar trend is also observed in the HER performance (Figure 22d). However, the coating hydrophilic polymers are often inactive so that too much polymer loaded on the surface of the electrocatalyst could lead to a significant loss in the OER or HER activity.

Nickel/cobalt‐based hydroxides have remarkable hydrophilic properties and catalytic activity, which is beneficial to promote HER and OER reaction kinetics.^[^ ^203^, ^205^ ^]^ Thus, introducing nickel/cobalt‐based hydroxides onto the surface of electrocatalyst could signally facilitate the improvement of electrolyte‐wettability and electrocatalytic performance. Xin and co‐workers coat a layer of amorphous Ni(OH)*_x_* onto Ni_3_S_2_ nanosheets through an electrochemical process to obtain a self‐supported Ni(OH)*_x_*/Ni_3_S_2_ heterostructure electrocatalyst on nickel foam (Ni(OH)*_x_*/Ni_3_S_2_/NF) (Figure 22e).^[^ ^203^ ^]^ The Ni(OH)*_x_*/Ni_3_S_2_/NF heterostructure electrocatalyst exhibits an excellent electrolyte‐wettable surface with a CA of 0°, which is obviously better than the pristine Ni_3_S_2_/NF with a CA of 146° (Figure 22f). The benefiting from the super electrolyte‐wettability, microporous feature, and self‐supported structure, the electrocatalyst exhibits an exceptional HER performance at large current density in alkaline electrolyte, only requiring low overpotential of 238 mV to reach a current density of 1000 mA cm^−2^ (Figure 22g) and displaying a long‐term durability up to 1000 h. Similar to the above effect, after the deposition of Ni(OH)_2_ on CoB by the wet chemical method, the shell–core structured Ni(OH)@CoB that Ni(OH)_2_ nanoflake is directly grown onto CoB nanochains has a much better electrolyte‐wettable than CoB, displaying the improved the OER catalytic performance with low overpotential and good OER durability.^[^ ^206^ ^]^

#### 4.2.2 Structural Designing for High Electrolyte‐Wettable Electrocatalyst

**Figure 23:**
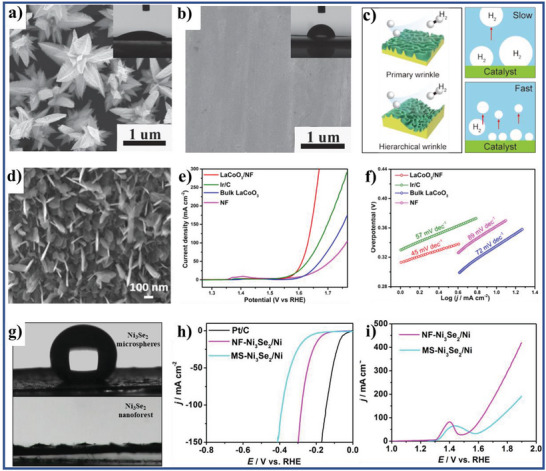
SEM image of a) pine‐shaped Pt nanostructured film and b) Pt flat film (the insets display the wettability of pine‐shaped Pt nanostructured film and Pt flat film to 0.5 m H_2_SO_4_ electrolyte. Reproduced with permission.^[^ ^207^ ^]^ Copyright 2015, Wiley‐VCH. c) Schematic illustration of different wrinkle structure and gas detachment from the surface during the HER. Reproduced with permission.^[^ ^208^ ^]^ Copyright 2019, American Chemical Society. d) SEM image of LaCoO_3_/nickel foam (NF), e) OER polarization curves, and f) Tafel slopes of LaCoO_3_/NF, bulk LaCoO_3_, Ir/C, and NF. Reproduced with permission.^[^ ^209^ ^]^ Copyright 2020, American Chemical Society. g) The CA of water on Ni_3_Se_2_ microspheres/Ni foil (top) and Ni_3_Se_2_ nanoforest/Ni foil (bottom). h) *I*–*R* corrected HER polarization curves of Ni_3_Se_2_ microspheres/Ni foil, Ni_3_Se_2_ nanoforest/Ni foil, and 20 wt% Pt/C on Ni foam (Pt/C), and i) OER polarization curves of NF‐Ni_3_Se_2_/Ni and MS‐Ni_3_Se_2_/Ni at a scan rate of 10 mV s^−1^ before *I*–*R* correction. Reproduced with permission.^[^ ^33^ ^]^ Copyright 2016, Elsevier.

In recent research, some Ni/Pt‐based metal/metal compounds tend to have higher surface energy with the better intrinsic electrolyte‐wettability.^[^ ^204^ ^]^ Based on Cassie mode and Wenzel mode, their electrolyte‐wettability will increase as the roughness increases. Thus, for HER and OER, metal/metal compound catalysts often use strategies of increasing surface roughness and designing special micro–nanostructures to alter wettability to electrolyte. The effect of different morphologies of Pt electrodes on electrolyte‐wettability and HER performance is studied by Li et al. It is found that the pine‐shaped Pt nanostructure electrode has ultralow water CA, compared with the Pt flat films, and the pine‐shaped Pt nanostructure electrode produces the smallest gas bubbles with the best HER performance (**Figure** **23**a,b).^[^ ^207^ ^]^ The phenomena are attributed to the array structure and ultrahigh roughness of the pine‐shaped Pt nanostructure, which prompts the rapid release of H_2_ bubbles and more active sites exposure with electrolytes. A series of hierarchical MoS_2_ with wrinkled structure is fabricated by chemical vapor deposition followed by wrinkling treatment.^[^ ^208^ ^]^ These hierarchical MoS_2_ wrinkles have improved electrolyte‐wettability relative to that of a primary wrinkle, which induces faster detachment of gas bubbles and results in enhanced HER performance compared with flat MoS_2_ and primary wrinkle MoS_2_ (Figure 23c). The same theory is most widely applied in OER and overall water splitting.^[^ ^35^, ^209^, ^210^ ^]^ Zhao et al. synthesize LaCoO_3_ perovskite oxides nanoarrays onto conductive nickel foam (LaCoO_3_/NF) by mild hydrothermal treatment followed by inert gas annealing.^[^ ^209^ ^]^ The porous structure LaCoO_3_/NF electrode formed by vertically aligned nanosheets (Figure 23d) increases surface roughness, thus enhancing electrolyte‐wettability of the surface. The proposed catalyst with enhanced electrolyte‐wettability displays an outstanding OER performance with lower overpotential of 342 mV at the current density of 10 mA cm^−2^ (Figure 23e) and the smallest Tafel slope of 45 mV dec^−1^ (Figure 23f).

A catalyst using for HER is usually active and stable at a certain PH value, but the PH value is not applicable to OER for the catalyst, which impedes the development of HER coupled with OER in an overall water splitting process for practical application.^[^ ^200^ ^]^ It is well known that some nickel chalcogenides with certain phases show excellent performance for OER and/or HER, separately.^[^ ^211^, ^212^ ^]^ Especially, the construction of electrolyte‐wettable surface is more conducive to the bifunctional electrocatalyst for overall water splitting, because electrolyte‐wettable surface could further lead to the effective contact of electrocatalyst with water and the bubble detachment from the electrode. The nanoforest Ni_3_Se_2_ with unique morphology and structure grown on Ni substrate (NF‐Ni_3_Se_2_/Ni) displays better electrolyte‐wettability than that of the Ni_3_Se_2_ microspheres grown on Ni substrate (MS‐Ni_3_Se_2_/Ni) in 1.0 m KOH (Figure 23g).^[^ ^35^ ^]^ When the electrolyte‐wettable NF‐Ni_3_Se_2_/Ni is used directly as the electrode for overall water splitting, it not only displays an excellent activity for HER with a required overpotential of 203 mV for 10 mA cm^−2^ (Figure 23h), but also is found to be higher active for OER with a required overpotential of 242 mV for 20 mA cm^−2^ (Figure 23i), which demonstrates that it has much better bifunctional electrocatalytic activity than that of the MS‐Ni_3_Se_2_/Ni.

#### 4.2.3 Surface Functionalization for Improving Electrolyte‐Wettability of Electrocatalyst

**Figure 24:**
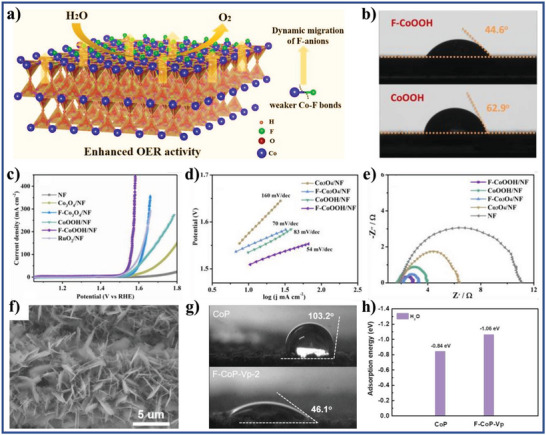
a) A fluorine‐anion surface engineering has been first put forward to activate catalytic active species of Co‐based materials, representing a completely new reconstruction way toward OER active species. b) Static water contact‐angle measurements of F‐CoOOH and CoOOH products. c) *IR*‐corrected OER polarization curves, d) Tafel slope, and e) electrochemical impedance spectra of FCoOOH/NF, F‐Co_3_O_4_/NF, CoOOH/NF, Co_3_O_4_/NF, RuO_2_/NF, and NF electrodes in 1 m KOH solution. Reproduced with permission.^[^ ^34^ ^]^ Copyright 2018, Wiley‐VCH. f) SEM image of the F‐CoP‐Vp NSs grown on carbon cloth. g) Static water CA measurements, and h) calculated water adsorption energy of CoP and F‐CoP‐Vp. Reproduced with permission.^[^ ^54^ ^]^ Copyright 2020, American Chemical Society.

Surface functionalization is a universal method of controlling the interfacial energy of a solid surface with electrolytes to realize the wettability tuning. Inspired by this, the functionalization surface of Fe/NF electrodes with borate reduces the interfacial energy between Fe sites and the electrolyte, which improves electrolyte‐wettability of the electrode and deceases the adhesion of gas bubbles.^[^ ^213^ ^]^ The electronegativity of F‐anion is strongest that will be much easier to form weak metal–F bonds with stronger ionicity (**Figure** **24**a), contributing to the dynamic migration of F‐anions and finally enriched on the surface of both cobalt (Co)‐based oxide/oxyhydroxide. After introducing F‐anions into CoOOH and Co_3_O_4_ products by a simple activation of electrochemical oxidation, surface enrichment of F‐anions endows smaller interfacial energy between the electrode and electrolytes and more electrolyte‐wettable surface character (Figure 24b) of F‐CoOOH and F‐Co_3_O_4_ electrode materials, which facilitates the contact between reactants and active sites, accelerates the key process of O‐related intermediates adsorption, enables the facile release of evolved O_2_ gas bubbles, and finally promotes the electron transfer to participate the following OER process (Figure 24a).^[^ ^34^ ^]^ The F‐CoOOH/NF electrode shows a lowest onset potential and overpotential (310 mV to reach the current density of 50 mA cm^−2^) (Figure 24c), highest current density, smallest Tafel slopes of only 54 mV dec^−1^ (Figure 24d), smallest *R*_ct_ (0.95 Ω) (Figure 24e) than that of unmodified CoOOH/NF and Co_3_O_4_/NF, indicating that the introduction of F anions has a significant improvement on OER reaction kinetics and OER catalytic activity under alkaline condition. F‐doped CoP nanosheet arrays (F‐CoP‐Vp NSs) (Figure 24f) also exhibit better HER catalytic activity, especially HER catalytic kinetics than that of the undoped sample in a nonacidic electrolyte, mainly ascribing to the improved electrolyte‐wettable surface (the water CA decreases from ≈103.2° to ≈46.1°, as shown in Figure 24g) and water adsorption energy of F‐CoP‐Vp (decreasing from −0.84 to −1.06 eV) (Figure 24h) deriving from the strong hydrogen bonding between F and water.^[^ ^54^ ^]^ The reaction kinetics of the electrodes can be compared by evaluating the Tafel slope and electrochemical impedance. The F‐CoP‐Vp NSs electrode shows the lowest Tafel slope of 88.9 mV dec^−1^ and the smallest *R*_ct_ value in neutral electrolyte. As a result, the optimized nanosheets array exhibits greatly enhanced HER catalytic activity in a neutral electrolyte. In addition, the electrolyte‐wettability of the electrode used in water splitting systems could also be enhanced by introducing hydrophilic groups such as hydroxyl group^[^ ^214^, ^215^ ^]^ and phosphate groups^[^ ^216^ ^]^ to promote the HER and/or OER performance.

## 5 Electrolyte‐Wettability of Electrode Materials in Capacitive Deionization

**Figure 25:**
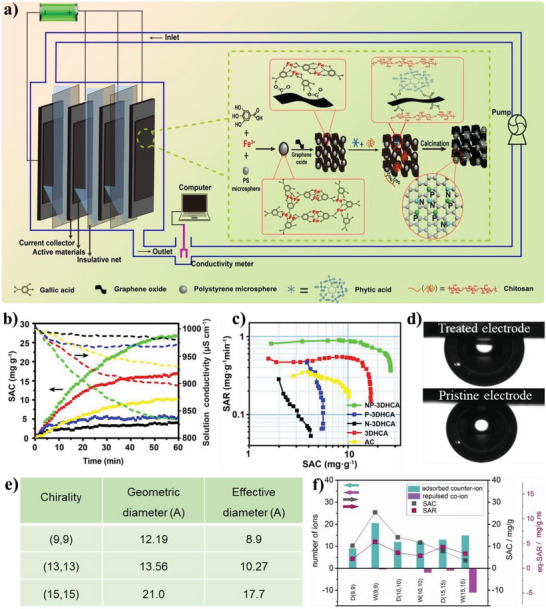
a) Schematic illustration of CDI device and preparation of N, P codoped 3DHCA. b) Plots of SAC versus time (the solid line) and solution conductivity versus time (the dot line) of different samples: NP‐3DHCA (the green line), P‐3DHCA (the red line), N‐3DHCA (the blue line), 3DHCA (the black line), and AC (the yellow line). c) Ragone plots of SAR versus SAC of different samples (all the above curves are measured at 1.2 V, 500 ppm, and 40 mL min^−1^). Reproduced with permission.^[^ ^36^ ^]^ Copyright 2018, The Royal Society of Chemistry. d) The changes in captive bubble CA of the O plasma treated carbon electrode (top) and the pristine activated carbon electrode (bottom). Reproduced with permission.^[^ ^217^ ^]^ Copyright 2013, Elsevier. e) The geometric and effective diameter of the SWCNT pores corresponding to different chiral indices and f) the number of electrosorbed/repulsed ions (left axis), SAC (right axis in black), and SAR (right axis in purple) for each of the SWCNT CDI systems (where the D and W initials are referring to the wettable and nonwettable states of the pores, respectively). Reproduced with permission.^[^ ^218^ ^]^ Copyright 2022, The Royal Society of Chemistry.

The work principle behind CDI is analogous to that of EDLCs. Driven by an external power supply, the charged ions are adsorbed by the reversely charged electrode under the influence of the electric field. Once the electric field is removed or reversed, the adsorbed ions are quickly released back to the bulk electrolyte, regenerating the CDI electrodes (**Figure** **25**a).^[^ ^36^, ^219^ ^]^ Carbon materials have received significant attention as electrode materials for CDI due to their high specific surface area, high conductivity, and good chemical inertness.^[^ ^36^ ^]^ However, it is well known that the nonpolar surface of pure carbon materials demonstrates inferior wettability with polar aqueous electrolyte, which reduces the utilization rate of high specific surface area of the carbon‐based CDI electrode materials, slows down electrolyte ions transfer into the inner channel of electrode, as well as shows a terrible CDI performance. N, P codoping (Figure 25a) could effectively improve the electrolyte‐wettability of carbon‐based electrode materials, further increasing their desalination capacity for removal of ions from saline water. The detailed CDI performances of NP‐3DHCA electrode are shown in Figure 25b,c. The NP‐3DHCA electrode displays the largest SAC of 26.8 mg g^−1^ after 60 min than that of other four samples (the SAC of P‐3DHCA, N‐3DHCA, 3DHCA, and AC is 16.6, 5.1, 3.6, and 10.2 mg g^−1^, respectively) (Figure 25b) and the highest SAR (Figure 25c) among the samples studied herein. Improving electrolyte‐wettability of carbon‐based CDI electrode could also be achieved by partially oxidizing the electrode active materials surface using O plasma and/or strong acid (HNO_3_ and H_3_PO_4_), owing to an increase in concentration of hydroxyl, carboxylic acid, ester groups, or phosphorus‐containing functional groups (such as P—O—C, P—OH, and P=O group) on the electrode surface after O plasma and/or strong acid treatment.^[^ ^217^, ^220^ ^]^ Compared to the pristine activated carbon electrode, the O plasma treated carbon electrode delivers a good electrolyte‐wettability (Figure 25d) and a higher SAC of 54.4 µmol g^−1^ (21.4% increase). With a view to the interactions between the O‐containing groups and —NH_3_^+^ and the polar —OH of water molecular, the grafted carbon electrodes by EDTA‐silane with abundant O‐containing groups and APTES with abundant —NH_3_^+^ exhibit improving electrolyte‐wettability and CDI performance (the removal efficiency is 99.9% at pH 6.0 for Pb^2+^ and 98.7% for Na^+^).^[^ ^221^ ^]^ A computational molecular dynamic study is used to investigate various desalination mechanisms of CDI systems with electrolyte‐wettable or electrolyte‐nonwettable armchair single‐wall carbon nanotube (SWCNT) electrodes with the chiral indices of 9, 10, and 15 as shown in Figure 25e.^[^ ^218^ ^]^ It is shown that the desalination mechanisms are directly influenced by the pore diameter and electrolyte‐wettability of nanotube electrode. The SAR, SAC, and the number of the exchanged ions of the nanotube electrodes are comparatively shown in Figure 25f.

The electrolyte‐wettability of nanotubes with a pore diameter larger than (9,9) nanotubes could improve the ionic mass exchange. However, this higher ionic exchange rate does not lead to higher desalination capacity and it results in lower desalination capacity due to the ion swapping and co‐ion repulsion. In the case of (9,9), the electrolyte‐wettability could increase the desalination capacity, which may be because the tight confinement and strong van der Waals interaction with the wall result in negligible co‐ion repulsion.

## 6 Summary and Perspectives

In conclusion, we summarize the recent development and application of electrolyte‐wettability of electrode materials. Initially, the basic electrolyte‐wettability mechanisms of electrode materials are introduced, such as definition of electrolyte‐wettability, typical electrolyte‐wettability models, Laplace pressure associated with electrolyte wetting, surface energy, and surface tension closely related to surface wettability, and measures to improve electrolyte‐wettability of electrode materials based on Young–Dupre theory. Second, the influence of electrolyte‐wettability of electrode materials on electrochemical energy storage performance of supercapacitors, metal ion batteries, and metal‐based batteries is described. In supercapacitors and metal ion batteries, the enhanced electrolyte‐wettability could increase ion‐accessible surface area of electrode materials, facilitate ion diffusion and transport in electrode/electrolyte interface and electrode materials internal, and improve ion movement and distribution in electrode/electrolyte interface, which results in increasing specific capacity, rate performance, energy density, and power density, and reducing electrochemical impedance, especially *R*_ct_. For metal‐based batteries, improving electrolyte‐wettability of anode could reduce nucleation overpotential, homogenize nucleation site, suppress preferential ion flux near dendritic tips, and form thin and stable SEI film, which renders dendrite‐free active metal deposition. The metal‐based batteries assembled by the electrode with excellent electrolyte‐wettability exhibit high energy density and power density, stable cycle performance, and good safety performance. However, some cathodes in metal‐based batteries, such carbon‐based O_2_ cathode and CO_2_ cathode, need remain moderate electrolyte‐wettability, because excessive electrolyte‐wettability is prone to result in the formation of flooding electrode, which negatively impacts the energy storage performance of the cathodes. Then, electrolyte‐wettability of electrode in electrochemical energy conversion system, such as fuel cells and electrochemical water splitting systems is discussed. Similar to the working atmosphere of O_2_ cathode and CO_2_ cathode, fuel cells generally present gas–solid–liquid three‐phase reaction system in the energy conversion process. Therefore, designing optimal balance of electrolyte‐wettability of electrode is critical to facilitate proton transport and prevent flooding, which can ameliorate high power performance and durability for fuel cells. Although electrochemical water splitting also occurs in gas–solid–liquid three‐phase reaction system, excellent electrolyte‐wettability is desired for the electrode. the electrode materials with excellent electrolyte‐wettable surface is beneficial to prompt effective contact of electrocatalyst with water and the bubble detachment, which facilitates the electron transfer and improves the capacity of water electrolysis. Finally, electrolyte‐wettability also significantly impacts electrochemical performance of the electrode materials used in CDI, because the work principle behind CDI is analogous to that of a EDLCs.

**Figure 26:**
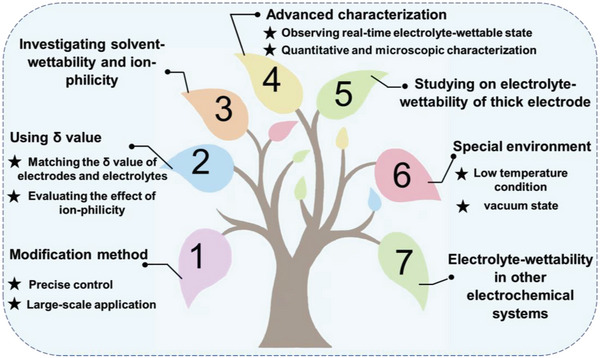
Future perspectives for electrolyte‐wettability of electrode materials.

Although significant progresses have been made in the study of electrolyte‐wettability of electrode materials, there are still some challenges in the related development research. In our view, the following seven points are worth noting (**Figure** **26**).

- The number of modification approaches that have been applied to improve electrolyte‐wettability of electrode materials is rising, but still open for significant growth, aided by the field of synthetic chemistry. Better control over the density, stability, and specific location of surface polar atom, functional groups, molecular or polymer brushes would be desirable. Besides, the large‐scale application of electrolyte‐wettable modification is still a huge challenge, especially for the introduction of surface functional groups, grafting molecular or polymer brushes. Some advanced technologies, such as plasma treatment and ultraviolet radiation may be considered as wettability modification methods for precise control and large‐scale application in the future.
- Solubility parameter (*δ*) as an indicator has been widely used to evaluate the interaction between polymer and solvent. However, based on the principle that the closer the *δ* is, the better the mutual wettability is, the design thought of matching *δ* between electrode material surface and electrolyte solvent is rarely used to improve electrolyte‐wettability of electrode materials. The matching *δ* could be performed either by adjusting the *δ* of electrode material surface or by adjusting the *δ* of electrolyte solvent. Furthermore, the *δ* of electrode material surface could be adjusted by introducing different functional groups, molecular brushes with different functional groups, or polymer brushes with different functional groups to be close to that of electrolyte solvent. In general, different functional groups show different ion‐philicity. Thus, the effect of ion‐philicity of electrode material surface on its macroscopic electrolyte‐wettability and electrochemical performance could be estimated by changing the ion‐philicity (namely, changing functional group introduced onto the electrode surface) while remaining the same *δ*.
- In the research of improving electrolyte‐wettability of the anode of metal‐based batteries (such as, Li metal anode, Na metal anode, K metal anode, Zn metal anode, and so on), the electrolyte‐wettability of anode should separate into solvent‐wettability and ion‐philicity to investigate their respective effects on electrochemical performance. When the anode has good solvent‐wettability, active metal is easy to contact with the electrolyte solvent, resulting in side reaction and consumption of the active metal, thereby rendering some negative effect on electrochemical performance of the anode.
- The characterization of electrolyte‐wettability of electrode materials is only qualitative, macroscopical, and no‐real‐time. A full understanding effect of the electrolyte‐wettability on the electrochemical performance of electrode materials requires using various characterization tools to cover a wide range of spatial (including phenomena, such as charge transfer, ion diffusion, the space charge layer, and physical contact) and temporal scales (including the speed of charge transfer and ion transport, and dynamic interface evolution). Specifically, more efforts should be devoted to investigating the interfacial electrolyte‐wettable state and real‐time interfacial changes of electrode materials in electrolyte under avoiding potential contaminations and artificial factors via some advanced analysis technologies, such as in situ scanning electron microscope (SEM), cryogenic SEM, cryo‐focused ion beam/scanning electron microscopy (cryo‐FIB/SEM) technologies, and new customizable technologies with integrated functions to enable information acquisition about the structure, composition, and kinetics.
- The use of thick electrodes is one of the effective measures to improve the volumetric performance of electrochemical energy storage devices, however, it has been found that the volumetric performance of many energy storage devices will not scale up linearly with the electrode thickness.^[^ ^222^ ^]^ One important reason is that the thick electrode has not good enough electrolyte‐wettability. Thus, the research on the electrolyte‐wettability of thick electrodes will have new scientific value and practical significance in the future.
- In the process of temperature reduction, not only the ionic conductivity of the electrolyte decreases, but also its surface energy and viscosity increase, and its fluidity become worse, which seriously reduces electrolyte‐wettability of electrode materials.^[^ ^40^ ^]^ Therefore, the design of electrolyte solvent may be preferred to improve the electrolyte‐wettability of electrode materials at low temperature. For example, some additives or miscible solvents could be used to reduce the viscosity and surface energy of the primary electrolyte at low temperature, as well as the freezing point of the primary electrolyte. Additionally, no reports have found on the study of electrolyte‐wettability of electrode materials in vacuum state maybe applied in aerospace electronics field.
- In addition to the six electrochemical systems mentioned in this paper, we propose that the influence of electrolyte‐wettability on electrochemical performance of electrode materials should be presented in all electrochemical systems with electrodes and liquid electrolytes, such as flow cells, electrochemical analysis and detection systems, photoelectrocatalysis systems, and so on, although the influence is not necessarily all positive.

## Conflict of Interest

The authors declare no conflict of interest.
